# Chasing Animals With Split Attention: Are Animals Prioritized in Visual Tracking?

**DOI:** 10.1177/2041669518795932

**Published:** 2018-09-03

**Authors:** Thomas Hagen, Thomas Espeseth, Bruno Laeng

**Affiliations:** Department of Psychology, University of Oslo, Norway; Department of Psychology, University of Oslo, Norway; NORMENT, Division of Mental Health and Addiction, Oslo University Hospital, Norway; Department of Psychology, University of Oslo, Norway

**Keywords:** animacy, animals, attention, animate monitoring hypothesis, multiple object tracking, multiple identity tracking, multiple event monitoring

## Abstract

Some evolutionary psychologists have hypothesized that animals have priority in human attention. That is, they should be detected and selected more efficiently than other types of objects, especially man-made ones. Such a priority mechanism should automatically deploy more attentional resources and dynamic monitoring toward animal stimuli than nonanimals. Consequently, we postulated that variations of the *multiple object or identity tracking* and *multiple event monitoring* tasks should be particularly suitable paradigms for addressing the animate monitoring hypothesis, given their dynamic properties and dependency on divided attention. We used images of animals and artifacts and found neither a substantial sign of improvement in tracking the positions associated with animal stimuli nor a significant distracting effect of animals. We also failed to observe a significant prioritization in orders of response for positions associated with animals. While we observed an advantage for animals in event monitoring, this appeared to be dependent on properties of the task, as confirmed in further experiments. Moreover, we observed a small but inconclusive advantage for animals in identity accuracy. Thus, under certain conditions, some bias toward animals could be observed, but the evidence was weak and inconclusive. To conclude, effect sizes were generally small and not conclusively in favor of the expected attentional bias for animals. We found moderate to strong evidence that images of animals do not improve positional tracking, do not act as more effective distractors, are not selected prior to artifacts in the response phase, and are not easier to monitor for changes in size.

## Introduction

According to the *animate monitoring hypothesis* (New, Cosmides, & Tooby, 2007), modern humans have inherited a perceptual mechanism that automatically monitors animate objects (i.e., humans and animals) or, in other words, items with high biological relevance (e.g., prey or predators). Such a mechanism should have provided our ancestors with the ability to quickly notice and keep track of nearby animals such that one could (adaptively) avoid becoming prey or miss an opportunity to hunt.

While the above hypothesis appears plausible from an evolutionary perspective, it is relevant to point out that a fundamentally similar distinction in the human semantic system between animate and inanimate (e.g., man-made artifacts like tools) has also been documented as patterns of selective impairments in neuroanatomically damaged patients (e.g., Capitani, Laiacona, Mahon, & Caramazza, 2003; Caramazza & Shelton, 1998; Gainotti, 2000, 2010; Hillis & Caramazza, 1991; Mahon & Caramazza, 2009; Warrington & Shallice, 1984). That is, in several neuropsychological studies, it has been shown that some patients can show a striking deficit for identifying animals while having a nearly intact ability to identify artifacts, whereas other patients show the reverse dissociation. Such findings are, however, not limited to patients, as a *normal* category-specific tendency in object identification has been observed in healthy individuals as well (Capitani, Laiacona, Barbarotto, & Trivelli, 1994; Låg, 2005; Låg Hveem, Ruud, & Laeng, 2006; Laws & Hunter, 2006; Laws & Neve, 1999). Furthermore, neurophysiological studies have shown a distinction between animate and inanimate objects in both humans and monkeys (e.g., inferotemporal response clustering of animate and inanimate objects; Kriegeskorte et al., 2008) and functional imaging have indicated similar distinctions (Konkle & Caramazza, 2013; Sha et al., 2015; Wiggett, Pritchard, & Downing, 2009), even in blind subjects without any prior visual experience (Mahon, Anzellotti, Schwarzbach, Zampini, & Caramazza, 2009).

While it remains unclear whether the neuropsychological observations described earlier stem from an innate or acquired distinction (e.g., Gainotti, 2015), they do suggest a strong relevance of visual and semantic classifications of animate and inanimate objects within, at least, the cognitive system of the primate brain. However, the animate monitoring hypothesis specifically proposes the presence of a low-level innate and adaptive mechanism for the classification of animate and inanimate objects. More specifically, according to New et al. (2007), animals should spontaneously and preferentially recruit more visual attention than artifacts regardless of their relevance to the task. Another critical aspect of the hypothesis is that, as animals in a natural setting can rapidly change their trajectory or position in a fraction of a second, the system should not only be geared toward detecting animals but also to actively monitor them in an ongoing manner through frequent inspections of their status (New et al., 2007). Accordingly, we expected that animals will bias the spatial distribution of attention and cause stronger spontaneous recruitment of attention. One area of category-specific attentional biases that have received some consideration and which, intuitively, is similar in nature to an attentional bias toward animals is the case of visual tracking of human faces (e.g., Li, Oksama, Nummenmaa, & Hyönä, 2017). Accordingly, one could anticipate that paradigms sensitive to human faces would also be sensitive to the presence of animals.

The seminal study by New et al. (2007) showed that animals were more readily detected in a *change detection* task (cf. Simons & Levin, 1997) where photographs containing animals and artifacts (man-made objects) would rapidly change, after a brief blank screen, so that an object would be briefly present or absent in quick succession while having its masked motion signal (see also Altman, Khislavsky, Coverdale, & Gilger, 2016). Crucially, the active monitoring aspect of the hypothesis is presumed to protect the animal stimuli from the effects of occlusion or interruptions of the visual scene. A wealth of studies have shown that the change in this task is not noticed immediately but takes time to be identified, as it is strongly dependent on the location of the focus of attention. Thus, the paradigm can be used fruitfully to evaluate the ability of specific items (or locations) to capture attention based on the lag between onset of the flickering image and a correct identification. Although New et al. (2007) gathered convincing evidence for an animacy advantage, such a finding has been questioned recently on methodological grounds (Hagen & Laeng, 2016; see also Hagen & Laeng, 2017), that is, it remains possible that the effect could stem from uncontrolled factors relating to aspects of the photographic scenes rather than of the object categories per se. This does of course not rule out that an attentional bias might actually exist as hypothesized and be measurable with other or more controlled tasks. In fact, recent research has shown that animate objects tend to be more easily localized in a visual search task (Jackson & Calvillo, 2013) as well as being more noticeable in an inattentional blindness task (Calvillo & Hawkins, 2016; Calvillo & Jackson, 2014). Hence, it seems important to explore in more depth the mechanisms involved and how they could relate to other aspects of attention. In particular, we reasoned that animacy, given that it is grounded in motion and dynamic action (Pratt, Radulescu, Guo, & Abrams, 2010), it should be especially relevant for dynamic aspects of attentional performance, that is, when visually tracking dynamic (moving) objects.

Indeed, in daily life, objects frequently change positions within our visual field, be it they change physical positions or because we move our body and eyes. Consequently, to monitor objects in our environment, we are taxed with the challenge of continuously and dynamically updating their positions. A task or experimental paradigm frequently used to study this ability is the *multiple object tracking* (MOT) task (e.g., Alnæs et al., 2014; Cavanagh & Alvarez, 2005), where participants are asked to track a subset of identical moving objects on a screen. While this paradigm was originally developed to study the limits of spatial and divided attention, it has also been used to study the binding of features and identities to the tracked objects (*what* and *where* dimensions; Cohen, Pinto, Howe, & Horowitz, 2011; Horowitz et al., 2007; Oksama & Hyönä, 2008; Pylyshyn, 2004) by assigning identities to objects or displaying them as unique objects in a multiple identity tracking (MIT) task. In particular, recent studies have indicated that certain categories or properties of the tracked objects can influence participants’ ability to keep track of objects’ identity and position. These studies have shown biases for stimuli such as angry faces (Li et al., 2017), attractive faces (Li, Oksama, & Hyönä, 2016; Liu & Chen, 2012), fearful faces (Jin & Xu, 2015), and some familiar objects (Oksama & Hyönä, 2008; Pinto, Howe, Cohen, & Horowitz, 2010). Moreover, results from a neuroimaging study that contrasted tracking of unique objects with tracking of a uniform set of objects have shown that temporal brain regions, typically associated with object recognition, are more engaged in the tracking of unique objects than in the tracking of a uniform set of objects (Nummenmaa, Oksama, Glerean, & Hyönä, 2017). Thus, performance on this task is sensitive to object content and appears to influence tracking ability beyond low-level features, suggesting that higher order processing can influence positional tracking. Given that the task is dependent on continuously distributed and divided attention and can be influenced by how the objects are individuated, it should be sensitive to a mechanism favored by natural selection for its ability to automatically and adaptively deploy sustained monitoring of the locations of animate objects (cf. New et al., 2007). Another recent development in this tracking paradigm is the *multiple event monitoring* (MEM) task (Wu & Wolfe, 2016) where participants are instructed to keep track of multiple moving images of objects while also monitoring them for changes. This task has to date not been employed to investigate attentional biases, but its requirement to actively monitor the status of objects for changes should be highly relevant for investigating the animate monitoring hypothesis.

As a bias toward automatically monitoring animals for changes in position or state should have had significant survival value for human ancestors, New et al. (2007) specified that the system did not just evolve to detect animals but also to autonomously monitor animals in an ongoing manner. In essence, the system should be sensitive to moving objects that looks like animals if these aspects played a role in the natural selection of the system.

We believe that being able to document an attentional bias toward animals with dynamic tracking tasks should be greatly beneficial to a further understanding of the extent or limits of animacy’s ability to influence dynamic, distributed, and sustained visual attention. Hence, the goal of this study is to attempt to document the presence and extent of such a bias. Specifically, estimates of effect sizes, a sufficient level of power, and Bayesian approaches appear to be necessary, as potentially nonsignificant results cannot be used as conclusive evidence for a particular effect being absent (Dienes, 2014). Furthermore, this approach should help narrowing down the set of situations where animate monitoring can have a sizable influence on the perceptual and attentional system.

More specifically, in line with the animate monitoring hypothesis, we expected to find that attention prioritizes animals in an automatic manner. Associating a task-relevant object with animacy (i.e., an image of an animal) should promote strong attentional allocation and vigilance toward that object and this effect should be measurable as prioritized responses as well as improved tracking and monitoring ability compared with objects that are not associated with animacy. Likewise, task-irrelevant animal distractors should be particularly capable to divert attention away from task relevant objects, which should be measurable as an increase in animal distractors being incorrectly reported as targets. In other words, participants should report animals more frequently than artifacts, irrespective of their status as targets or distractors.

## Experiment 1

One model of how tracking takes place in MOT proposes that the observer allocates one focus of attention per target (Cavanagh & Alvarez, 2005) and, consequentially, a limited pool of neural resources gets divided among them (Alnæs et al. 2014; Kahneman, 1973). The aim of the present, initial, experiment was to attempt to influence this assignment process by presenting one of the targets as an animal. According to the animate monitoring hypothesis, animals should automatically capture attention more strongly than other objects. Hence, our hypothesis was that targets displayed as animals during the target assignment phase in an MOT task would bias the amount of resources assigned to it and thereby result in improved tracking accuracy. In addition, we predicted that this process of prioritization should also lead to a bias in the order in which targets are reported, and that animal distractors should be reported as targets more frequently than artifact distractors.

More specifically, in the first experiment, two randomly positioned images of objects were used as targets while another set of 10 objects were used as distractors. After viewing the targets for a period of time sufficient to identify each of them uniquely, we occluded the objects with black disks during the tracking period such that the images were only visible during target assignment. We made the straightforward prediction that animal targets would be tracked more successfully than artifact targets. Second, as participants were free to report the targets in any order they liked, we predicted that animal targets would be reported (clicked on) before targets presented as artifacts due to the presumed prioritization process. Third, as targets’ localization errors would be dependent on target–distractor confusions, we predicted that when participants made erroneous responses, that is, a distractor was reported as a target, it would be more likely for such a distractor to be an animal rather than an artifact. This was expected from the supposedly automatic prioritization of animals in attention, which should make it more likely for targets’ *swaps* (Drew, Horowitz, & Vogel, 2013) to be biased toward animal distractors (i.e., targets should be more confusable with animal distractors than artifact distractors). To probe this, we computed the *percentage of incorrect responses* per participant, as the percentage of animal and artifact distractors (across all trials) that were selected during the response phase.

Power analysis based on an estimated mean $\eta_{p}^{2}$ of .3 from previous studies (Jin & Xu, 2015; Li et al., 2016, 2017; Liu & Chen, 2012) indicated that at least 22 participants were required to obtain 80% power in detecting a 4% advantage in accuracy for animals (*d_z_* = 0.65, *d_rm_* = 0.40). However, to encompass an even smaller advantage of 2.5%, we aimed to test at least a twofold sample of about 50 participants ($\eta_{p}^{2}$ = .15, *d_z_* = 0.40, *d_rm_* = 0.25). Some of the experiments in the original study by New et al. (2007) were designed to have 80% power to detect an effect size of *d_z_* = 0.47. Although no specific reasoning for this level of sensitivity has been provided, this may serve here as an indication of the theorists’ intended minimum effect size of interest. At any rate, our experiment should be able to detect an effect size smaller than the minimum implied by the existing reports in favor of the animate monitoring hypothesis. In fact, we should be able to detect an effect size of a magnitude much smaller than what has been typically detected in studies on attentional biases (Jin & Xu, 2015; Li et al., 2016, 2017; Liu & Chen, 2012; New et al., 2007).

To be able to quantify the evidence for and against a given hypothesis, we used JASP (https://jasp-stats.org/) to calculate Bayes factors (BFs; with JASP’s default prior). We report BF_01_ in favor of the null hypothesis, expressing the probability of the data given the null hypothesis relative to the alternate hypothesis (e.g., a value of 7 would suggest that the observed data are seven times more likely to have occurred under the null hypothesis than under the alternate hypothesis). Specifically, as the value of BF_01_ increases above 1, there is more evidence in support of the null hypothesis (e.g., that an effect is likely to be absent). Conversely, as the value decreases below 1, there is more evidence in support of the alternate hypothesis (e.g., that an effect is likely to be present). Inverting BF_01_ (1/BF_01_) yields BF_10_, which expresses how likely the data are under the alternate hypothesis relative to the null hypothesis (Dienes, 2014; Jarosz & Wiley, 2014). The BFs can be further interpreted or categorized based on the obtained value, for example, a BF_01_ in the range 1 to 3 (or BF_10_ within 1–0.33) can be viewed as anecdotal (i.e., weak, inconclusive) evidence (see Andraszewicz et al., 2015; Wetzels, Ravenzwaaij, & Wagenmakers, 2015, for the interpretations adopted here).

### Methods

#### Participants

We recruited 68 (20 women) participants with a mean age of 33 years (range: 18–57 years, standard deviation [*SD*] = 8.81 years). All were recruited with *Crowdflower®*. All participants agreed to an informed consent approved by the institute’s internal review board (Reference No. 1439337) and in accordance with the Declaration of Helsinki.

#### Apparatus

The experiment was implemented with JavaScript and each participant ran the experiment on their own computer, as is typically the case with crowdsourcing experiments (Crump, McDonnell, & Gureckis, 2013).

#### Stimuli

We used the Snodgrass and Vanderwart’s (1980) stimulus set of line drawings to select 20 animal and 20 artifact images that were balanced on complexity ratings, animals: *M* = 3.76, *SD* = 0.48, artifacts: *M* = 3.62, *SD* = 0.46, *t*(37.98) = 0.91, *p* = .366, familiarity ratings, animals: *M* = 2.66, *SD* = 0.84, artifacts: *M* = 2.87, *SD* = 0.71, *t*(36.98) = 0.83, *p* = .414, and number of pixels, animals: *M* = 1,684, *SD* = 460, artifacts: *M* = 1,804, *SD* = 550, *t*(36.85) = 0.75, *p* = .456. The images of animals were the following: alligator, ant, bird, cat, chicken, cow, deer, dog, donkey, elephant, fox, gorilla, horse, mouse, peacock, pig, rabbit, rooster, snake, and squirrel. The images of artifacts were the following: airplane, baby carriage, barn, barrel, bicycle, cannon, church, French horn, gun, helicopter, iron, motorcycle, rocking chair, roller skate, sailboat, trumpet, violin, wagon, watch, and whistle. These images were then used to generate MOT trials with one animal and one artifact as targets and five animals and five artifacts as distractors. A pool of 240 unique paths were used to select paths for each participant in the following way: First, we selected 30 random paths and assigned images to them before we copied the paths and images while switching the category of the targets to create 30 new paths. Thus, balancing object movements across target categories so that any difference between categories is not attributable to a set of paths being easier for one category than for another, as they were identical. The repetition of paths should not be of significant concern with regard to improving performance by chance for one specific category, as trial orders were randomized across participants and a large number of repetitions would be typically required to improve performance considerably (Ogawa, Watanabe, & Yagi, 2009). This setup resulted in 60 experimental trials per participant.

#### Procedure

**Figure 1.**
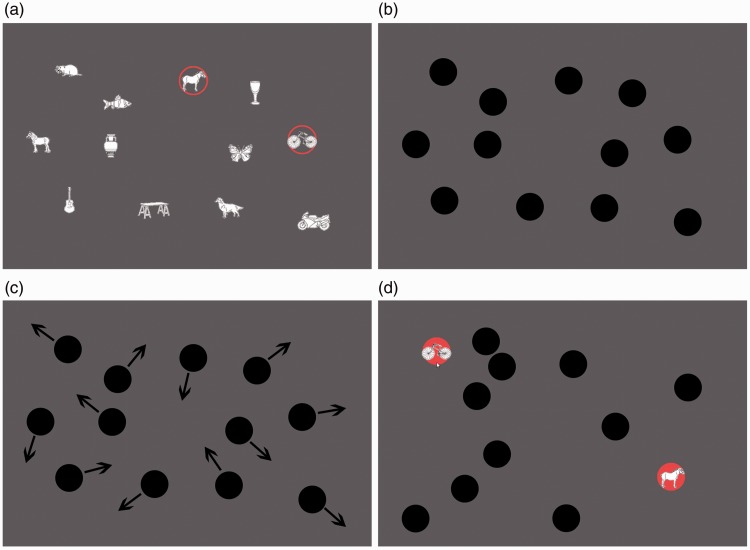
Illustration of a trial in Experiment 1. First, the targets were indicated by enclosing them in red circles (a), then all objects were hidden behind black disks (b) before they started moving around the screen (c). Participants indicated the positions of the targets when their movement stopped by clicking on them (d), which also made the objects visible, to provide feedback.

The task started with the presentation of 12 randomly positioned and nonoverlapping objects for 200 milliseconds before two of them were designated as targets by enclosing them in red circles for 1,500 milliseconds (see Figure 1(a)). Next, the red circles flashed for 1,500 milliseconds before the objects were occluded by black disks and started moving around the screen. The tracking period lasted for a random duration between 5 and 7 seconds, but durations were the same between identical paths. The display was redrawn at a rate of 30 frames per second, and the objects moved with a speed of 16 pixels per frame in a display measuring 1,200 × 800 pixels. The displays were scaled dynamically to encompass differences in screen resolutions by resizing the display area to fit within the browser window of devices not supporting the full resolution. Participants were instructed to click on the target objects as soon as their movement stopped. Feedback was given by indicating the number of correctly identified objects. Each participant was required to complete five practice trials with at least 75% correct prior to starting the experiment. The practice trials contained a different set of images than those used in the main experiment (Op de Beeck & Wagemans, 2001). Task instructions were presented with textual stepwise descriptions and illustrations as well as a video demonstration of the task.

### Results

**Figure 2.**
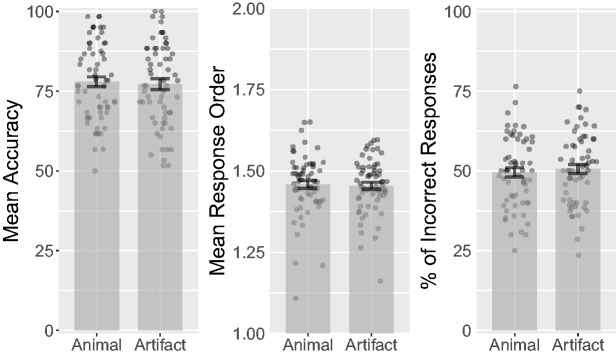
Combined bar and scatter plots on mean accuracy, response orders (lower numbers indicate earlier responses), and percentage of incorrect responses (distractors reported as targets) over target category in Experiment 1 (i.e., percentages of animal and artifact distractors that were selected during the response phase). Error bars show standard errors, and the superimposed scatterplots show mean values of each participant.

Before conducting the statistical analysis, we removed data from five participants for having a mean accuracy that was below 50% (1.5 *SD* below the median). A *t* test on tracking accuracy between the two categories showed no significant difference in accuracy between animal and artifact targets (see Figure 2), *t*(62) = 0.9, *p* = .37, 95% confidence interval (CI) [−0.91, 2.4], *d_z_* = 0.11, *d_rm_* = 0.058, *common language* (*CL*) *effect size* = 55% (see Lakens, 2013, for reported effect size estimates). Animal targets had a mean accuracy of 77.9% (*SD* = 12.1%), while artifact targets had a mean accuracy of 77.2% (*SD* = 13.2%). A Bayesian paired samples *t* test showed moderate evidence (Wetzels et al., 2015) for the null hypothesis, BF_01_ = 4.93, suggesting that our data are 4.93 more likely to be observed under the null hypothesis than the alternate hypothesis.

To investigate whether participants reported animal targets before artifact targets, we conducted a *t* test on mean response orders, which showed no significant difference, *t*(62) = 0.26, *p* = .8, 95% CI [ − 0.024, 0.032], *d_z_* = 0.033, *d_rm_* = 0.041, CL = 51% (see Figure 2). Animal targets had a mean value of 1.46 (*SD* = 0.096), while artifact targets had a mean value of 1.45 (*SD* = 0.084). Response orders ranged from 1 to 2, where 1 would represent the first response a subject made on a trial, while 2 would represent the last response. A Bayesian *t* test showed moderate evidence for the null hypothesis, BF_01_ = 7.012.

To investigate whether animal distractors were reported as targets more frequently than artifact distractors, we conducted a *t* test on percentage of incorrect responses (distractors reported as targets) between animals (*M* = 49.5%, *SD* = 11%) and artifacts (*M* = 50.5%, *SD* = 11%). Two participants were removed from this analysis for having only one incorrect response. The result showed no significant difference, *t*(60) = 0.37, *p* = .71, 95% CI [−6.8, 4.7], *d_z_* = 0.047, *d_rm_* = 0.095, CL = 52% (see Figure 2). A Bayesian *t* test showed moderate evidence in favor of the null hypothesis, BF_01_ = 6.68.

### Discussion

The present results did not reveal statistically significant support for the hypothesis that presenting objects as animals and artifacts during the target assignment phase in an MOT task, should lead to (a) improved tracking accuracy for animal targets, (b) earlier responses for animal targets, or (c) more animal distractors being reported as targets. Similarly, the BFs consistently showed moderate support for the null hypothesis of no effect of images of animals across measures. Given these results, it seems unlikely that our measures are substantially different between the two types of images used. As with any experimental report, the research community should decide whether the potential for even smaller effect sizes than what our study was powered for is deemed interesting and worthwhile pursuing in larger samples.

One reason for the present findings could be that simply presenting targets as animals during assignment is not sufficient to evoke a measurable bias. That is, the hypothesis rests on the assumption that prioritization is assigned to a token location of an animal and that this can be maintained as long as an attentional locus is assigned to the object, irrespective of the fact that the object no longer depicts the figure that could lead to such prioritization. Although several studies with MOT provide evidence that the visual system can track such items independent of their original identity (e.g., color), it is possible that the supposed attentional bias assigned to targets cannot be easily maintained as the objects turn to black disks and change positions during the several seconds of the tracking phase. Essentially, the presented images during the target assignment phase might have been mostly irrelevant to participants and, perhaps consequentially, became irrelevant for tracking performance as well. Hence, in the next experiment, we maintained the visibility of the images.

To prevent ceiling performance in this task, we had set a speed deemed to be sufficient for yielding errors. One possibility is that such a relatively high speed was not appropriate to uncover an advantage for animals. Thus, in the next experiment, we increased the number of targets while lowering the speed in an attempt to cause more competition between multiple attentional foci and thus induce stronger priority.

## Experiment 2

The first experiment failed to indicate any attentional biases toward animals. One possibility is that the previous experiment did not pose enough competition between attentional foci to bring about an advantage for animals. According to New et al. (2007), high levels of focused attention should reduce the impact of task-irrelevant nonanimals more than task-irrelevant animals. Thus, using four targets and lowering the speed of the objects (so as to not make the task too difficult), we aimed to make it more relevant for the attentional system to perform prioritizations. Moreover, most previous studies using MOT or MIT to investigate attentional biases have used three to six targets (Jin & Xu, 2015; Li et al., 2016, 2017; Liu & Chen, 2012).

In addition, most studies demonstrating improved tracking performance for particular categories of objects (Li et al., 2017; Liu & Chen, 2012) have kept the objects visible during the tracking phase. Such an experimental design may also be more suited to test the animate monitoring hypothesis, as it specifically proposes that animals should be monitored continuously, in an ongoing manner. This may presuppose that their shape is visible while they are tracked, at least for most of the time. Besides, in ecological conditions, we rarely track a subset of identical objects that previously had a visible identity and then lost it. Hence, we generated a version of the task that would seem closer to natural scenarios. For completeness, a version with the objects hidden during tracking was also conducted at an early stage of the study and is included as Supplementary Experiment 1.

As for the previous experiments, the main prediction was that animals would be tracked more successfully as targets than artifacts. Second, we predicted that animal targets would be reported before artifact targets. Third, we predicted that when participants made erroneous responses, it would be more likely for an animal distractor to be reported as a target than for an artifact distractor. Conceivably, the present design should be more sensitive to this aspect, as the continuously visible animals could grab attention at any time during tracking.

### Methods

#### Participants

We recruited 67 participants (13 women) with a mean age of 32.3 years (range: 19–59 years, *SD* = 9.52 years).

#### Stimuli

We used the same images as in Experiment 1.

#### Procedure

**Figure 3.**
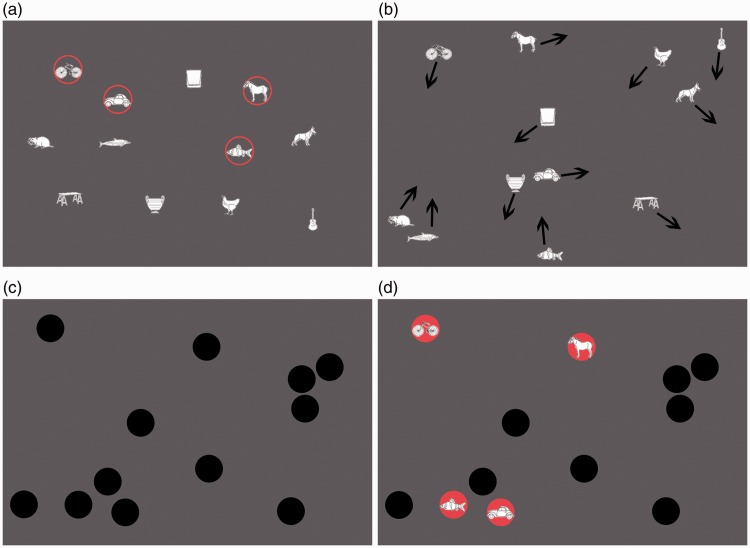
Illustrationof a trial in Experiment 2. Objects were visible during assignment (a) and tracking (b) but hidden at the last frame (c) before the response phase (d).

This was similar to Experiment 1, except for the following: We used four targets (two animals and two artifacts), lowered the speed to 12 pixels per frame to avoid making the task too difficult, and objects were visible during the tracking period but hidden on the last frame before the movements stopped (see Figure 3(c)). Combined with a variable trial duration, the change in procedure aimed to avoid a strategy of simply remembering how the targets looked in order to report them correctly (i.e., they were required to continuously keep track of them).

### Results

**Figure 4.**
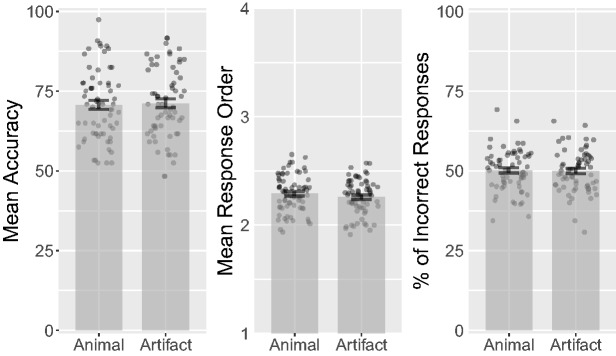
Combined bar and scatter plots for Experiment 2 on mean accuracy, response orders, and percentage of incorrect responses by category. Error bars show standard errors, and the superimposed scatterplots show mean values from each participant.

Before performing statistical analysis, we removed three participants for having a mean accuracy below 50% (1.5 *SD* below the median). A *t* test on accuracy between animal (*M* = 70.7%, *SD* = 11.2%) and artifact (*M* = 71.1%, *SD* = 11.2%) targets showed no significant difference, *t*(63) = 0.4, *p* = .69, 95% CI [−2.5, 1.7], *d_z_* = 0.049, *d_rm_* = 0.037, CL = 52% (see Figure 4). A Bayesian *t* test showed moderate evidence for the null hypothesis, BF_01_ = 6.77.

In contrast to the previous experiment, response orders ranged from 1 to 4, where 1 would represent the first response a subject made on a trial, while 4 would represent the last response. A *t* test on response order between animal (*M* = 2.29, *SD* = 0.18) and artifact (*M* = 2.26, *SD* = 0.16) targets also showed no significant difference, *t*(63) = 1.4, *p* = .15, 95% CI [−0.012, 0.073], *d_z_* = 0.18, *d_rm_* = 0.18, CL = 57%. A Bayesian *t* test showed anecdotal evidence for the null hypothesis, BF_01_ = 2.702.

Then, to investigate whether animal distractors were reported as targets more frequently than artifact distractors, we applied a *t* test on percentage of incorrect responses (distractors reported as targets) between animals (*M* = 50.1%, *SD* = 6.6%) and artifacts (*M* = 49.9%, *SD* = 6.6%). Again, this analysis also showed no significant difference, *t*(63) = 0.11, *p* = .91, 95% CI [−3.1, 3.5], *d_z_* = 0.014, *d_rm_* = 0.027, CL = 51% (see Figure 4). A Bayesian *t* test showed moderate evidence for the null hypothesis, BF_01_ = 7.256.

### Discussion

Despite increasing the number of targets and continuously displaying the target objects as animals and artifacts during tracking, we did not observe significantly more accurate tracking of target animals as compared with artifact targets. In addition, in line with the previous experiment, we failed to observe a significant precedence in reporting of animal targets in this experiment as well. Finally, we also failed to observe significantly more frequent erroneous reporting of animal distractors compared with artifacts. Despite the fact that each trial was structured to induce competition between animal and artifact attentional foci in the presence of hypothetically attention-grabbing animal distractors, we failed to reject any of the null hypotheses. In fact, the obtained BFs showed moderate evidence for the null hypothesis for the measures on accuracy and percentage of incorrect responses. However, the BF for response orders was only anecdotal, which does not warrant a firm conclusion on its support for the null hypothesis. Rather, the data appear insensitive in distinguishing between the null and the alternative hypothesis for the measure on response orders (Dienes, 2014).

In summary, it appears that artifacts can be tracked just as efficiently as animals, and we found no conclusive evidence for prioritizations of either category, nor did we observe that animate objects were conclusively more effective in capturing attention in target–distractor confusions, even though the images were continuously visible. Again, researchers should decide whether the potential for even smaller effect sizes than what our study was designed for is deemed relevant.

## Experiment 3

In the previous couple of experiments, the identity of the objects was mostly irrelevant to the task; thus in the following experiment, we made the identity of the objects explicitly relevant, using image probes during the response phase, while requiring participants to localize them. Previous work using such probes has indicated more successful tracking of object properties presumed able to induce attentional biases (i.e., attractive faces and emotional expressions: Jin & Xu, 2015; Li et al., 2016; Liu & Chen, 2012). Moreover, using such image probes during the response phase, we required participants to be aware, at all times, of which objects were tracked and where these were located.

According to the animate monitoring hypothesis, this type of explicit requirement should not be necessary for observing a bias toward animals, as the bias is supposed to behave in an automatic way regardless of current goals. However, as the previous experiments failed to bring about a substantial advantage for animals, we reasoned that the situation constructed here could increase the chance of revealing such a bias.

We expected that in such conditions, the binding and tracking of animal targets would be more successful than for artifacts. Thus, the task is similar to Experiments 1 and 2, except for making the appearance of the objects at assignment directly relevant for performance. Due to the extensive literature on category-specific deficits for animals in naming, recognition and memory (e.g., Capitani et al., 1994; Låg, 2005; Låg et al., 2006; Laws & Hunter, 2006; Laws & Neve, 1999; Nairne, VanArsdall, & Cogdill, 2017; Nairne, VanArsdall, Pandeirada, Cogdill, & LeBreton, 2013), it seems difficult to purely attribute an effect of superior identity tracking accuracy for animals as stemming from an attentional bias. Consequently, we designed for the acquisition of position accuracy measures as well, by making the task sufficiently difficult, so as to avoid ceiling effects.

**Figure 5.**
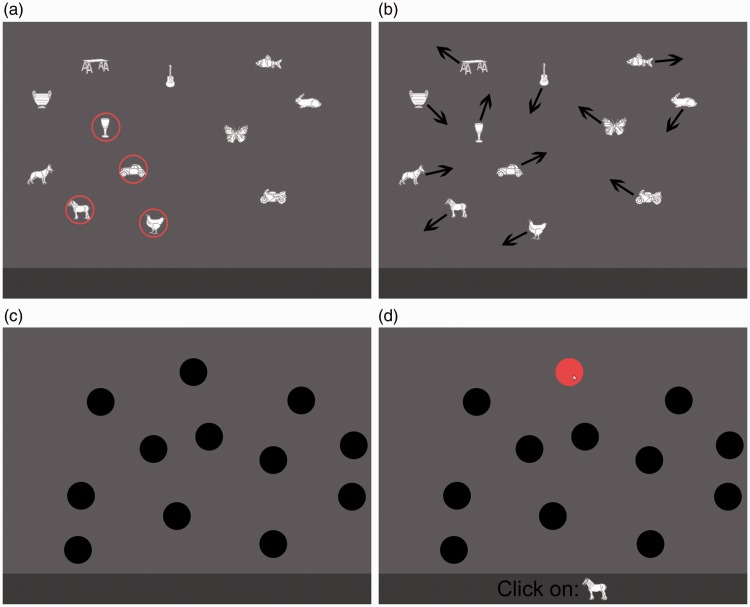
Illustration of a trial in Experiment 3. First targets were assigned by enclosing them in red circles (a), then all objects started moving around the display (b) before being hidden at the last frame when the movements stopped (c). Probes appeared at the bottom of the display during the response phase (d), where participants indicated the position of the probes.

The design of this study allowed for investigating both identity tracking performance and position tracking performance. We defined identity accuracy as the percentage correct localizations of the probe images displayed at the bottom of the screen (see Figure 5). We defined position accuracy as the percentage correct localizations of targets, irrespective of their identities. The expectation was that the identity of animal targets would be tracked more successfully than the identity of artifact targets and, consequently, we expected their positions to be tracked more successfully as well. As in the previous experiments, we also predicted that animal distractors would be reported as targets more frequently than artifact distractors.

Another version of this experiment, kept the objects hidden during tracking in an attempt to make the bindings between identity and positions more volatile. We included this experiment as Supplementary Experiment 2.

### Methods

#### Participants

We recruited 71 participants (21 women) with a mean age of 32.2 years (range: 18–57 years, *SD* = 8.3 years).

#### Stimuli

We used the same set of images as in the previous experiments.

#### Procedure

This was similar to Experiment 2, with the exception that participants were required to indicate the position of the objects displayed at the bottom of the screen in the response phase (see Figure 5(d)). Identity accuracy was based on how accurately participants could localize the individual targets after the tracking period, while position accuracy was defined as how accurately target positions were reported irrespective of their identity. Each target was probed sequentially in a counterbalanced manner between animals and artifacts. The circles turned red when clicked on during the response phase. The incorrect and correct objects were revealed along with feedback about accuracy once the required number of objects had been reported.

### Results

Before conducting the statistical analysis, we removed the data from five participants for having mean identity accuracy below 35% (1.5 *SD* below the median). A *t* test on identity accuracy showed no significant difference between animal (*M* = 59.4%, *SD* = 14.4%) and artifact (*M* = 57.4%, *SD* = 13.5%) targets, *t*(65) = 1.3, *p* = .19, 95% CI [−1, 5.1], *d_z_* = 0.16, *d_rm_* = 0.14, CL = 56%. A Bayesian *t* test showed moderate evidence for the null hypothesis, BF_01_ = 3.238.

**Figure 6.**
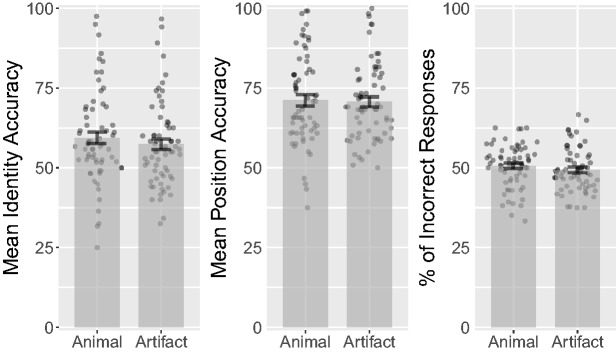
Combined bar and scatter plots for Experiment 3 on mean identity accuracy, position accuracy, and percentage of incorrect responses by category. Error bars show standard errors, and the superimposed scatterplots show mean values from each participant. Identity accuracy shows how accurately participants could localize the individual targets after the tracking period. Position accuracy shows percentage correct localizations of targets, irrespective of their identities.

A further *t* test on position accuracy showed no significant difference between animal (*M* = 71.2%, *SD* = 14.1%) and artifact (*M* = 70.7%, *SD* = 12.2%) targets, *t*(65) = 0.29, *p* = .78, 95% CI [−2.5, 3.4], *d_z_* = 0.035, *d_rm_* = 0.032, CL = 51% (see Figure 6). A Bayesian *t* test showed moderate evidence for the null hypothesis, BF_01_ = 7.121.

Before analyzing the percentage of incorrect responses on distractors by category, we removed two participants for having five or less incorrect responses. A *t* test between animals (*M* = 50.6%, *SD* = 6.8%) and artifacts (*M* = 49.4%, *SD* = 6.8%) showed no significant difference, *t*(63) = 0.74, *p* = .46, 95% CI [−2.1, 4.7], *d_z_* = 0.092, *d_rm_* = 0.18, CL = 54%. A Bayesian *t* test showed moderate evidence for the null hypothesis, BF_01_ = 5.627.

### Discussion

Although the images were continuously visible throughout tracking and participants were explicitly required to track their identities, we failed to observe statistically significant advantages for animal targets over artifact targets in identity and position accuracy as well as in percentages of incorrect responses. Consistently, the obtained BFs showed moderate evidence for the null hypothesis of no effect of images of animals. It thus seems unlikely that our measures were substantially different between the two types of images used.

An alternative design could have required participants to locate the objects by name rather than image, which could in turn have promoted a strategy for encoding more semantic aspects of the objects. However, according to the animate monitoring hypothesis, explicit semantic processing of animals should not be required for obtaining an attentional advantage.

## Experiment 4

Some MIT variants in previous studies with facial stimuli have used designs where targets and distractors are either from the same or from the different categories (Jin & Xu, 2015; Li et al., 2016, 2017; Liu & Chen, 2012). Such a design allows for the simultaneous testing of differences in the ability of the categories in holding and attracting attention. Moreover, this design allows participants to separate targets and distractors categorically in a subset of trials, which may relax the need to relay on object identity during tracking. While it may not be clear why this arrangement should be more sensitive to an attentional bias for animals than our previous attempts, our primary motivation was to use a design that has had history of successfully demonstrating biases to categories of objects.

An underlying assumption in these studies is that the binding of an identity, which may have an associated attentional bias, to its position, should improve tracking performance of that position, as it moves around the display, independently of the explicit requirement of tracking its identity (Li et al., 2017). Despite this apparent assumption, most previous studies have focused on the acquisition of identity accuracy measures. In fact, the majority of studies with facial stimuli and identity probes did not analyze position accuracies due to ceiling effects (Li et al., 2016, 2017), but one study reported an advantage for fearful over neutral faces in both position tracking accuracy and identity tracking accuracy (Jin & Xu, 2015). Thus, we specifically designed the experiment to obtain position accuracies as well. With this setup, we predicted that animals would yield an advantage in both identity accuracy and position accuracy. Moreover, we predicted that animal distractors would lead to more errors than artifact distractors.

### Methods

#### Participants

We recruited 67 participants (17 women) with a mean age of 33 years (range: 18–67 years, *SD* = 10 years).

#### Stimuli

We used the same set of images as in the previous experiments.

#### Procedure

Similar to Experiment 3, except that targets were either four animals or artifacts, while distractors were either eight animals or artifacts.

### Results

Before performing the statistical analysis, we removed four participants for having mean identity accuracy below 35% (1.5 *SD* below the median). An analysis of variance (ANOVA) on identity accuracy over target category (animal, artifact) and distractor category (animal, artifact) revealed a significant main effect of target category, *F*(1, 62) = 5.15, *p* = .027, $\eta_{p}^{2}$ = .08, $\eta_{g}^{2}$ < .01. There was, however, neither a significant effect of distractor category, *F*(1, 62) = 1.34, *p* = .251, $\eta_{p}^{2}$ = .02, $\eta_{g}^{2}$ < .01, nor a significant interaction, *F*(1, 62) = 3.83, *p* = .055, $\eta_{p}^{2}$ = .06, $\eta_{g}^{2}$ < .01. Animal targets had a mean accuracy of 56.2% (*SD* = 13.6%), while artifact targets had a mean accuracy of 54.4% (*SD* = 14%). Trials with animal distractors had a mean accuracy of 55.6% (*SD* = 14%), while trials with artifact distractors had a mean accuracy of 54.9% (*SD* = 13.7%). A Bayesian repeated measures ANOVA on identity accuracy revealed anecdotal evidence that target category had an effect, BF_01_ = 0.49, and moderate evidence for the null hypothesis for distractor category, BF_01_ = 4.5.

**Figure 7.**
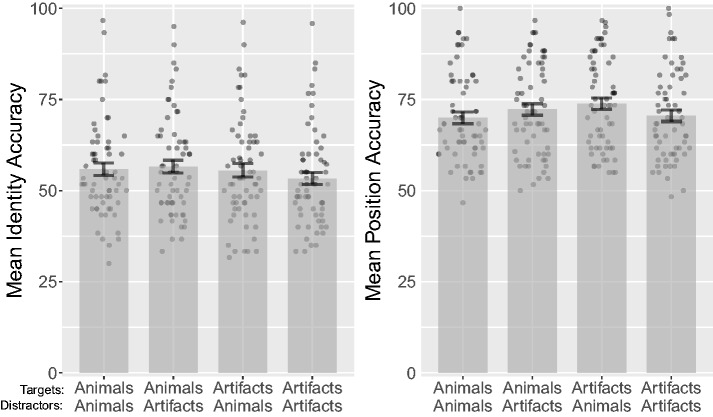
Combined bar and scatter plots for Experiment 4 on mean identity and position accuracy by target category and distractor category. Error bars show standard errors, and the superimposed scatterplots show mean values from each participant. Identity accuracy shows how accurately participants could localize the individual targets after the tracking period. Position accuracy shows percentage correct localizations of targets, irrespective of their identities.

An ANOVA on position accuracy over target category and distractor category revealed a significant main effect of target category, *F*(1, 62) = 4.90, *p* = .030, $\eta_{p}^{2}$ = .07, $\eta_{g}^{2}$ < .01, but not distractor category, *F*(1, 62) = 0.83, *p* = .366, $\eta_{p}^{2}$ = .01, $\eta_{g}^{2}$ < .01. The interaction effect was also significant, *F*(1, 62) = 13.50, *p* < .001, $\eta_{p}^{2}$ = .18, $\eta_{g}^{2}$ < .01 (see Figure 7). Animal targets had a mean accuracy of 71.1% (*SD* = 12.6%), while artifact targets had a mean accuracy of 72.1% (*SD* = 12.6%). The reverse of the identity results; animal targets leading to reduced position accuracy performance. Trials with animal distractors had a mean accuracy of 71.9% (*SD* = 12.6%), while trials with artifact distractors had a mean accuracy of 71.4% (*SD* = 12.6%), implying that artifacts were slightly more effective distractors than animals. A Bayesian repeated measures ANOVA on position accuracy revealed anecdotal evidence for the null hypothesis for target category, BF_01_ = 1.97, as well as moderate evidence for the null hypothesis for distractor category, BF_01_ = 5.61.

### Discussion

In line with predictions, the identity of animal targets was reported significantly more successfully than the identity of artifact targets. However, contrary to the prediction that a similar advantage should be found in position accuracy, the results showed that artifacts were tracked significantly more successfully than animals. Based on these tendencies, it would seem that participants were better at tracking the identity of animals but not their positions. Suggesting that participants were slightly better at remembering where they saw a particular animal. In addition, we found no significant effect of animal distractors, which is in line with previous studies (Li et al., 2016, 2017) as well as our previous experiments. The obtained BFs for identity accuracy were mostly in line with the significance tests. However, the BFs helped to reveal that the evidence for the alternate hypothesis of target category was only anecdotal (Wetzels et al., 2015). Thus, we cannot conclusively state that animal identities were tracked better than artifact identities. The BFs also helped to cast doubt on the statistically significant result of target category in position accuracy by showing that the null hypothesis was 1.97 times more likely than the alternate hypothesis given the data. Thus, the results indicated anecdotal evidence for no difference between the categories in position accuracy.

Although the Bayesian results did not warrant any conclusion with regard to the effect of category on identity and position accuracies, it is still interesting to consider that the indicated patterns of results might not necessarily be attributed to attention. As the results indicated that participants were not better at tracking positions associated with animals but were better at remembering what they depicted, this might suggest an advantage in memory (Nairne et al., 2013, 2017) or encoding (Hagen & Laeng, 2017). Indeed, more effective encodings of animals from brief exposures (as implied by the brief target inspections occurring in such tasks, Oksama & Hyönä, 2016) might yield the indicated advantage in reporting where particular animals were localized. Specifically, a recent study with rapid presentations of animals (Hagen & Laeng, 2017) showed that animal targets were encoded more successfully for later report than artifacts but still did not gain prioritized access to attention.

Finally, it must be stressed that the observed effect sizes in the present experiment are relatively small and far from what should be expected from the original account (New et al., 2007). In fact, the Bayesian analysis indicated that the effects of category on identity and position accuracies were weak and inconclusive. Thus, future studies should aim to test a larger sample if the potential for such small effects are deemed interesting and worthwhile.

## Experiment 5

While the previous experiments largely failed to observe any clear attentional biases for animals, the animate monitoring hypothesis specifically proposed that the mechanism evolved to monitor the location and state of animate objects. Consequently, the features offered by the change detection task were deemed important by the original investigators of the hypothesis (New et al., 2007). The investigation thus far has probed more the aspect of keeping track of the changing positions of animals, but we have not yet assessed the importance of actually monitoring the state of objects. It is also possible that the original change detection design (New et al., 2007) lacked some dynamic aspects which the mechanism may be particularly sensitive to, considering that it evolved in a dynamic and noisy world (e.g., animals may be moving about, but only certain aspects of their translations in space is relevant for behavior). Another aspect of the change detection task is that it relies on disrupting visual processing by blanking the screen to mask changes in state, which may have unknown influences on the putative monitoring system. Thus, the concept of combining an MOT task with a change detection task appears to have merits worthy of an investigation despite the apparent lack of evidence so far from either type of paradigms (e.g., Hagen & Laeng, 2016).

Such combinations have been attempted in unrelated investigations (Bahrami, 2003; Oksama & Hyönä, 2008), relying on invasive disruptions of visual processing (blanking and mud splashes). A recent development, however, is the MEM task, where participants are required to continuously monitor the state of multiple objects moving randomly around a display (Wu & Wolfe, 2016). The task is thus similar to the MOT or MIT tasks, with the notable exception that participants are to monitor all objects for a specific change and respond as fast as possible when a change occurs. Importantly, in this paradigm, the objects are continuously visible as changes in state occur. Because changes in state can induce visual transients drawing attention to their location, the paradigm relies on small clockwise and counterclockwise rotations, of each stimulus throughout the duration of the tracking period, to mask the signal from a single transitory change in state, enforcing participants to pay close attention to the state of objects rather than relying on a transient signal from one of the objects. In the version of the task presented here, we changed the state of objects by manipulating their lateral (horizontal) orientation at a random point in time. This task thus combines the continuous distributed and dynamic attention aspects of the MOT or MIT paradigms with a change detection task requiring participants to respond as fast as possible when they detect a change in state of animals and artifacts. This task should thus be more similar to the type of task thought to be sensitive to the attentional bias for animals (New et al., 2007), that is, engaging active monitoring of the location and state of animals while imposing vigilance toward responding to changes that are relevant for behavior.

The main prediction was that participants should detect changes to animals faster and more correctly than changes to artifacts. In addition, we used two levels of load, as previous research with this paradigm (Wu & Wolfe, 2016) has indicated that only about two to three objects can be tracked successfully (Wu & Wolfe, 2016); we decided to have trials with two or four objects. If humans’ typical tracking capacity is two to three objects, then tracking four objects should presumably help in bringing forward an advantage for animals, especially if these are prioritized in attention.

### Methods

#### Participants

For Experiment 5A, we recruited 60 participants (24 women) with a mean age of 31 years (range: 17–64 years, *SD* = 9.75 years). For Experiment 5B, we recruited 55 participants (8 women) with a mean age of 30 years (range: 17–67 years, *SD* = 10.7 years).

#### Stimuli

For experiment 5A we selected 20 animals (alligator, ant, bear, cow, donkey, fish, fly, frog, gorilla, horse, kangaroo, lobster, monkey, mouse, penguin, pig, rabbit, rooster, seal, and snake) and 20 artifacts (airplane, baby carriage, bicycle, three cars, church, digger, gun, rocking chair, scooter, stroller, Swiss army knife, teapot, telescope, tractor, triangle ruler, trumpet, watering can, and whistle) that we judged to have clear directionality across six sets of line drawings (Bates et al., 2003; Bonin, Peereman, Malardier, Méot, & Chalard, 2003; Cycowicz, Friedman, Rothstein, & Snodgrass, 1997; Op de Beeck & Wagemans, 2001; Nishimoto, Miyawaki, Ueda, Une, & Takahashi, 2005; Snodgrass & Vanderwart, 1980). The groups of objects were matched on degree of visual change they would induce by matching them on the number of pixels that would vary when they changed orientation from left to right, *t*(37.509) = 0.21, *p* = .83, as well as number of pixels (size), *t*(36.168) = 0.32, *p* = .75. For Experiment 5B, we selected, from the same sources as Experiment 5A, 20 animals (ant, bird, bug, bull, cat, deer, dove, fawn, lion cub, monkey, mouse, raccoon, rhinoceros, sea horse, seagull, seal, spider, turtle, and two wolves) and 20 artifacts (ambulance, beer mug, camper, canon, car, caravan, two drills, flipper, grenade, hook, key, mixer, pipe, saw, stapler, traffic light, tricycle, violin, and wheelbarrow). The stimuli were also matched on how much they would change by being flipped (mirror flipped horizontally), *t*(37.648) = 0.13, *p* = .89, and number of pixels, *t*(34.41) = 0.28, *p* = .77.

#### Procedure

**Figure 8.**
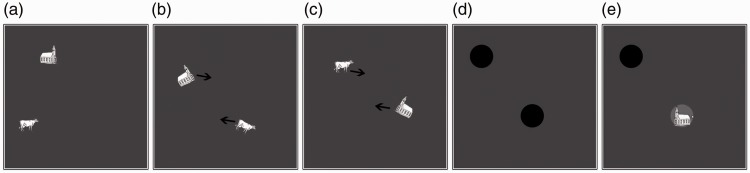
Illustration of the procedure in Experiment 5. First, targets were assigned (a), then all objects started moving around the display while frequently rotating 30° left and right (b), a change in lateral orientation occurred at a random time point between 2 and 6 seconds from start of tracking (c; notice that the church changes lateral orientation, as signaled by the position of the belfry). The movements stopped once a total of 8 seconds had elapsed or the observer pressed a button. Black disks immediately occluded the objects as the movements stopped (d). Participants then indicated the position of the changed object (e). Images adapted with permission (Snodgrass & Vanderwart, 1980, pp. 197–204).

Each trial started with the presentation of a set of objects for 3 seconds. Next, the movement phase started and the objects moved randomly around the screen for 8 seconds (see Figure 8), or until response. The objects moved with a speed of 8 pixels per frame (30 frames per second) in a display measuring 800 × 800 pixels. Objects were also randomly tilted by 30° to the left or right for short durations (233 milliseconds) in order to mask any unique transients imposed by the change in orientation of the target object (Wu & Wolfe, 2016). Participants were instructed to look for a change in lateral orientation in any of the objects and press the space bar, as quickly as possible, to indicate that they had detected a change in orientation. Black disks immediately occluded the objects once the button had been pressed. Participants were then instructed to indicate the target by clicking on it with the mouse pointer. If they failed to press the button before 8 seconds had elapsed, the trial ended with the objects occluded and the participant guessed which one had changed. A change in orientation always happened within the time range of 2 to 6 seconds. Thus, there was a sufficient time to detect a change before the trial ended. Each trial contained an equal number of animals and artifacts. The experiment included 80 trials, divided over target category (animal, artifact) and number of objects (load 2, load 4). All trial movements were randomly generated for each participant, but movement patterns were matched between the target categories. Participants were required to complete six practice trials where at least four were correct and responded to within 2 seconds of the change.

### Results

#### Experiment 5A

**Figure 9.**
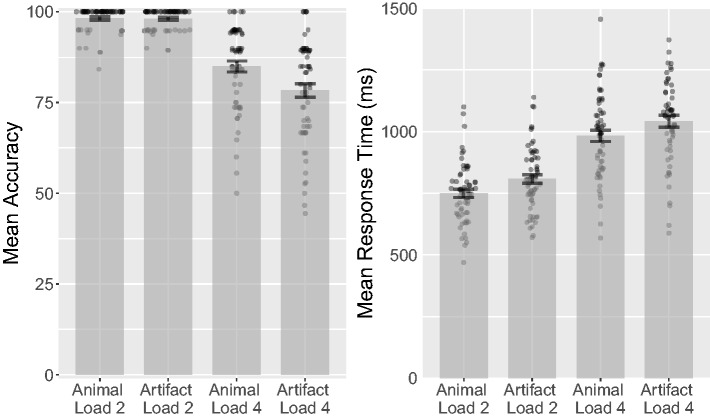
Mean accuracy and RTs in Experiment 5A. Error bars show standard errors.

To analyze accuracy, we ran an ANOVA on Category (animal, artifact) and Load (Load 2, Load 4), which showed significant main effects of Category, *F*(1, 59) = 12.71, *p* < .001, $\eta_{p}^{2}$ = .18, $\eta_{g}^{2}$ = .03, and Load, *F*(1, 59) = 149.29, *p* < .001, $\eta_{p}^{2}$ = .72, $\eta_{g}^{2}$ = .43. The interaction of the two factors was also significant, *F*(1, 59) = 13.91, *p* < .001, $\eta_{p}^{2}$ = .19, $\eta_{g}^{2}$ = .03. At Load 2, both categories had near ceiling performance, as animals had a mean accuracy of 98.2% (*SD* = 3.62%) and artifacts had a mean accuracy of 98.1% (*SD* = 3.03%), *t*(59) = 0.15, *p* = .88, 95% CI [ − 0.83, 0.97], *d_z_* = 0.019, *d_rm_* = 0.02, CL = 51%. At Load 4, animals had a mean accuracy of 85% (*SD* = 11.6%), while artifacts had a mean accuracy of 78.3% (*SD* = 14.4%), *t*(59) = 3.8, *p* = .00039, 95% CI [3.1, 10], *d_z_* = 0.49, *d_rm_* = 0.5, CL = 69% (see Figure 9). A Bayesian repeated measures ANOVA revealed anecdotal evidence for the alternate hypothesis for Category, BF_01_ = 0.84, and extreme evidence for the alternate hypothesis of Load, ${\text{BF}}_{01}=1.71\times 10^{-31}$.

For the analysis of response times (RTs), we selected trials in which a correct response was made within 2,000 milliseconds from a change in orientation. An ANOVA on RT over Category (animal, artifact) and Load (Load 2, Load 4) showed significant main effects of Category, *F*(1, 59) = 20.48, *p* < .001, $\eta_{p}^{2}$ = .26, $\eta_{g}^{2}$ = .03, and Load, *F*(1, 59) = 230.86, *p* < .001, $\eta_{p}^{2}$ = .80, $\eta_{g}^{2}$ = .37. Their interaction was not significant, *F*(1, 59) < 0.01, *p* = .996, $\eta_{p}^{2}$ < .01, $\eta_{g}^{2}$ < .01. At Load 2, animals had a mean RT of 749.7 milliseconds (*SD* = 124.1 milliseconds), while artifacts had a mean RT of 808 milliseconds (*SD* = 132.7 milliseconds), *t*(59) = 5.5, *p* < .001, 95% CI [−80, − 37], *d_z_* = 0.71, *d_rm_* = 0.45, CL = 76%. At Load 4, animals had a mean RT of 984.2 milliseconds (*SD* = 174.8 milliseconds), while artifacts had a mean RT of 1,042 milliseconds (*SD* = 182 milliseconds), *t*(59) = 2.5, *p* = .013, 95% CI [−100, −13], *d_z_* = 0.33, *d_rm_* = 0.33, CL = 63%. A Bayesian repeated measures ANOVA revealed moderate evidence for the alternate hypothesis for Category, BF_01_ = 0.272, and extreme evidence for alternate hypothesis of Load, ${\text{BF}}_{01}=4.06\times 10^{-35}$.

#### Experiment 5B

**Figure 10.**
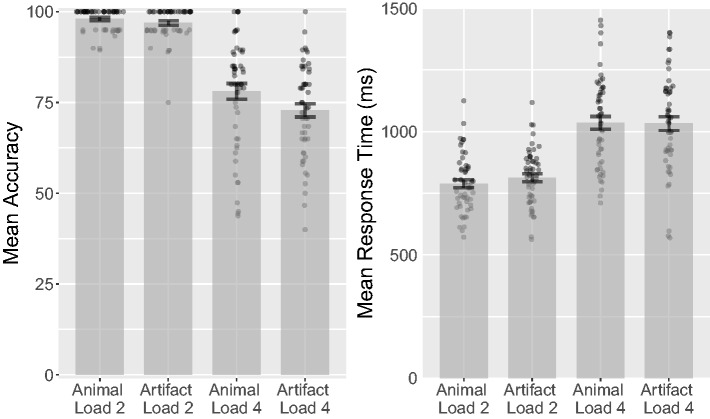
Mean accuracy and RTs in Experiment 5B. Error bars show standard errors.

Before the analysis, we removed three participants for having mean accuracy below 65% (1.5 *SD* below the median). For the analysis of accuracy, we ran an ANOVA on Category (animal, artifact) and Load (Load 2, Load 4), which showed significant main effects of Category, *F*(1, 51) = 8.27, *p* = .006, $\eta_{p}^{2}$ = .14, $\eta_{g}^{2}$ = .02, and Load, *F*(1, 51) = 175.82, *p* < .001, $\eta_{p}^{2}$ = .78, $\eta_{g}^{2}$ = .53. Their interaction was also significant, *F*(1, 51) = 4.24, *p* = .045, $\eta_{p}^{2}$ = .08, $\eta_{g}^{2}$ < .01. At Load 2, animals had a mean accuracy of 98% (*SD* = 3.1%), while artifacts had a mean accuracy of 96.9% (*SD* = 4.57%), *t*(51) = 1.3, *p* = .19, 95% CI [−0.56, 2.8], *d_z_* = 0.19, *d_rm_* = 0.29, CL = 57%. At Load 4, animals had a mean accuracy of 78.1% (*SD* = 15.6%), while artifacts had a mean accuracy of 72.8% (*SD* = 13%), *t*(51) = 2.7, *p* = .0093, 95% CI [1.3, 9.1], *d_z_* = 0.37, *d_rm_* = 0.36, CL = 65% (see Figure 10). A Bayesian repeated measures ANOVA revealed anecdotal evidence for the null hypothesis for Category, BF_01_ = 2.145, and extreme evidence for the alternate hypothesis for Load, ${\text{BF}}_{01}=1.9\times 10^{-36}$.

An ANOVA on RT over Category (animal, artifact) and Load (Load 2, Load 4) showed that the main effect of Category was not significant, *F*(1, 51) = 0.48, *p* = .491, $\eta_{p}^{2}$ < .01, $\eta_{g}^{2}$ < .01, and that the main effect of Load was, however, significant, *F*(1, 51) = 150.07, *p* < .001, $\eta_{p}^{2}$ = .75, $\eta_{g}^{2}$ = .36. Their interaction was not significant, *F*(1, 51) = 1.00, *p* = .322, $\eta_{p}^{2}$ = .02, $\eta_{g}^{2}$ < .01. At Load 2, animals had a mean RT of 788.7 milliseconds (*SD* = 117.3 milliseconds), while artifacts had a mean RT of 813.5 milliseconds (*SD* = 116.1 milliseconds), *t*(51) = 1.8, *p* = .07, 95% CI [−52, 2.1], *d_z_* = 0.26, *d_rm_* = 0.21, CL = 60%. At Load 4, animals had a mean RT of 1,036 milliseconds (*SD* = 183.7 milliseconds), while artifacts had a mean RT of 1,033 milliseconds (*SD* = 198.9 milliseconds), *t*(51) = 0.098, *p* = .92, 95% CI [−51, 56], *d_z_* = 0.014, *d_rm_* = 0.014, CL = 51%. A Bayesian repeated measures ANOVA revealed moderate evidence for the null hypothesis for Category, BF_01_ = 5.62, and extreme evidence for the alternate hypothesis for Load, ${\text{BF}}_{01}=1.16\times 10^{-27}$.

### Discussion

The results from Experiment 5A revealed that changes to animals were reported significantly more accurately and faster than changes to artifacts. This seems to be in agreement with the animate monitoring hypothesis (New et al., 2007) and to bring support to the idea that the act of monitoring objects for changes is an important aspect for observing a bias for animals. However, there is a possibility that the animal stimuli were somehow easier to monitor for changes than artifacts due to some uncontrolled factors pertaining to the chosen set of images. It is thus interesting to consider the results from Experiment 5B, which used a different set of images. Similar to the previous experiment, this experiment also appeared to reveal significantly more accurate reporting of changes to animals as compared with artifacts, but it did not replicate the observation of faster detections of animal changes. While the BF for the effect of category on accuracy was in agreement with the significance results of Experiment 5A, it did not agree with the significance results of Experiment 5B. The significance tests of Experiment 5B showed that animals were tracked significantly more accurately than artifacts, while the BF showed anecdotal evidence for the null hypothesis for the same data. Given this set of results and the fact that we have only anecdotal evidence for the alternate and null hypothesis across experiments (5A, 5B), the results on the effect of category on accuracy appear inconclusive. The evidence for an effect of category in RT was in fact moderate in both experiments, with Experiment 5A showing evidence for the alternate hypothesis while Experiment 5B showing evidence for the null hypothesis. Thus, we are here faced with a conflicting set of results.

In summary, both experiments were inconclusive in relation to an effect of category on accuracy. Experiment 5A showed moderate evidence for an effect of category on RT, while Experiment 5B showed moderate evidence for no effect of category on RT. Specifically, the effect of images of animals on accuracy and RT would appear not to be robust or of considerable size, as well as appearing to be dependent on the stimuli used. While it is possible that humans are more sensitive to changes in lateral orientation of animals, such a requirement appears too specific in relation to the general advantage for animals we are seeking to find (New et al., 2007). In addition, we cannot rule out the effect some uncontrolled low-level aspects that somehow made the monitoring of the animals’ lateral orientation easier (e.g., that a protruding head and neck pointing in a certain direction could be easier to detect to have changed than objects not suggesting such directionality). Thus, it would seem appropriate to attempt to generalize the indications observed here to another type of change. Another type of change that might seem even more relevant in a survival scenario is changes in size. A change in size would intuitively signal that an animal is either getting closer or further away from the viewer, a situation which intuitively should be more relevant for survival than animals turning left and right. Thus, the next experiment was designed to directly address whether the advantage is dependent on the type of change participants were monitoring for.

## Experiment 6

To assess whether the indication of an advantage for monitoring animals in Experiment 5 is specific to lateral (horizontal) orientation changes or can be generalized to another type of change that would be perhaps even more relevant in a survival scenario, we selected changes in size as another type of change to monitor for. Intuitively, changes in size provide visual cues for apparent distance of an object from an unmoving viewer so that a size-changing object may appear to move in depth during the change.

As for the previous experiment, it is predicted that changes to animals would be detected more accurately and faster than changes to artifacts. Similar to the previous experiment, we also chose to conduct a replication in a parallel experiment (6B) with a different set of images.

If the advantage for animals in Experiment 5 was related to an animate monitoring bias, then we would expect to find analogous effects in the present experiment. Conversely, if the animal advantage was specific to lateral changes and not related to an animate monitoring advantage, we expected no marked advantage for animals.

In addition, we checked the post hoc hypothesis that increases in size should cue for increased proximity (e.g., looming; Schiff, Caviness, & Gibson, 1962) and thereby be more salient and pertinent to yielding an animal advantage than decreases in size.

### Methods

#### Participants

For Experiment 6A, we recruited 71 participants (10 women) with a mean age of 28 years (range: 18–58 years, *SD* = 7.49 years). For Experiment 6B, we recruited 64 participants (13 women) with a mean age of 31.2 years (range: 18–63 years, *SD* = 8.71 years).

#### Stimuli

**Figure 11.**
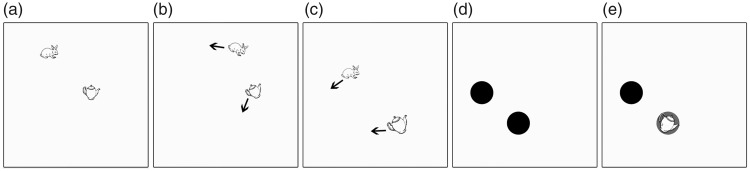
Example of a trial in Experiment 6. First, targets were assigned (a), then all objects started moving around the display while frequently rotating 30° left and right (b), a change in size occurred (larger or smaller) at a random time point between 2 and 6 seconds from start of tracking (c; notice that the teapot increases in size). The movements stopped once a total of 8 seconds had elapsed or the observer pressed a button. The objects were immediately occluded behind black disks as the movements stopped (d). Participants then indicated the location of the changed object (e). Image of rabbit adapted with permission (Snodgrass & Vanderwart, 1980, pp. 197–204).

For Experiment 6A, we used the same set of images as in Experiment 5A. As this set was balanced on the number of pixels that would change in lateral inversions, it was not balanced on the amount of change introduced by changing their sizes. Thus, we resized the images such that the mean overall size, *t*(37.98) = 0.03, *p* = .973, number of dark pixels (darker than 0.6 on a gray scale from 0 to 1), *t*(33.5) = 0.81, *p* = .4, and amount of change in overall pixels, *t*(37.95) = 0.05, *p* = .957, and dark pixels, *t*(30.224) = 1.07, *p* = .3, introduced by scaling them 25% would not be significantly different between animals and artifacts. Part of this process involved matching individual images of animals and artifacts on size and amount of change such that they would be as similar as possible on those measures. The resulting pairs were then used in trial generation in which 40 unique sets of movement patterns (20 sets with two objects and 20 sets with four objects) were randomly selected for each participant from a pool of 240 paths. Then, for generating trials, an equal number of animals and artifacts were assigned to each set of movement patterns before target animals, change times, initial size (±12.5%), and type of change (implied by initial size, as initially small objects would increase in size while initially large objects would shrink in size by 25%) were assigned in a counterbalanced manner. For the artifact trials, we simply duplicated the animal trials to create another set of 40 trials but replaced animals with artifacts and artifacts with animals. To make the changes in size less conspicuous, we also changed the background to white (see Figure 11).

For Experiment 6B, we selected, from a pool of 61 animals and 80 artifacts, 20 new pairs of animals (bear, beetle, bug, bull, camel, cow, duck, kangaroo, moose, mouse, parrot, rabbit, raccoon, seahorse, shark, snail, swan, turtle, and two wolves) and artifacts (bathtub, bicycle, flipper, harmonica, kitchen knife, kite, lamp, light bulb, motorbike, mousetrap, purse, rocking chair, skateboard, stroller, table, teapot, telescope, tractor, violin, and watering can). The new set were chosen, as it was more appropriate for balancing on visual properties deemed important with regard to size changes (number of pixels changing when resized; in contrast, the set selected for, e.g., Experiment 5B was balanced on number of pixels that changed when flipped left and right). The images were resized to be minimally different in number of overall pixels, *t*(38) = 0.02, *p* = .982, dark pixels, *t*(38) = 0.06, *p* = .95, and amount of change in overall pixels, *t*(38) = 0.02, *p* = .985, and dark pixels, *t*(37.975) = 0.015, *p* = .99, introduced by scaling them 25%. The trial generation process was identical to Experiment 6A.

#### Procedure

The overall procedure was identical to Experiment 5, with the exception that participants were instructed to report when one of the objects changed size. The change in size was either 25% smaller or larger.

### Results

#### Experiment 6A

**Figure 12.**
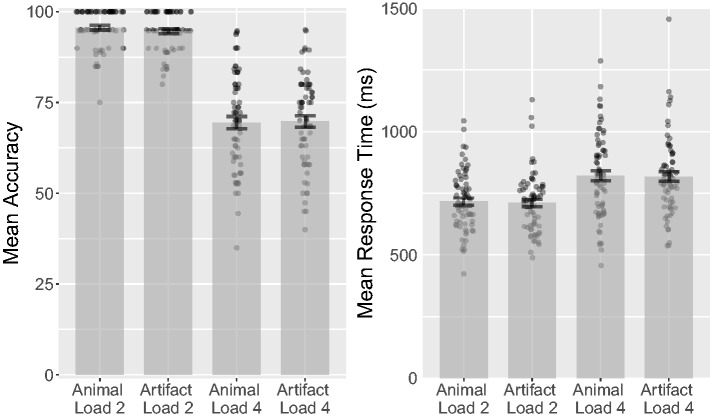
Mean accuracy and RTs in Experiment 6A. Error bars show standard errors.

Before analyzing the data, we removed three participants for having mean accuracy below 65% (1.5 *SD* below the median). An ANOVA on Category (animal, artifact) and Load (Load 2, Load 4) showed a nonsignificant main effect of Category, *F*(1, 67) = 0.11, *p* = .742, $\eta_{p}^{2}$ < .01, $\eta_{g}^{2}$ < .01, but a significant main effect of Load, *F*(1, 67) = 535.97, *p* < .001, $\eta_{p}^{2}$ = .89, $\eta_{g}^{2}$ = .62. The interaction was not significant, *F*(1, 67) = 0.40, *p* = .530, $\eta_{p}^{2}$ < .01, $\eta_{g}^{2}$ < .01. At Load 2, animals had a mean accuracy of 95.6% (*SD* = 5.51%), while artifacts had a mean accuracy of 94.6% (*SD* = 5.46%), *t*(67) = 1.2, *p* = .23, 95% CI [ − 0.66, 2.7], *d_z_* = 0.15, *d_rm_* = 0.18, CL = 56%. At Load 4, animals had a mean accuracy of 69.5% (*SD* = 13.6%), while artifacts had a mean accuracy of 69.8% (*SD* = 12.6%), *t*(67) = 0.15, *p* = .88, 95% CI [−4.1, 3.6], *d_z_* = 0.018, *d_rm_* = 0.022, CL = 51% (see Figure 12). A Bayesian repeated measures ANOVA revealed moderate evidence for the null hypothesis for Category, BF_01_ = 7.31, and extreme evidence for the alternative hypothesis for Load, ${\text{BF}}_{01}=8.5\times 10^{-63}$.

An ANOVA on RT showed a nonsignificant main effect of Category, *F*(1, 67) = 0.14, *p* = .707, $\eta_{p}^{2}$ < .01, $\eta_{g}^{2}$ < .01, and a significant main effect of Load, *F*(1, 67) = 66.66, *p* < .001, $\eta_{p}^{2}$ = .50, $\eta_{g}^{2}$ = .12. The interaction was not significant, *F*(1, 67) = 0.02, *p* = .883, $\eta_{p}^{2}$ < .01, $\eta_{g}^{2}$ < .01. At Load 2, animals had a mean RT of 717.1 milliseconds (*SD* = 121.7 milliseconds), while artifacts had a mean RT of 710.8 milliseconds (*SD* = 122.1 milliseconds), *t*(67) = 0.49, *p* = .63, 95% CI [−19, 32], *d_z_* = 0.059, *d_rm_* = 0.052, CL = 52%. At Load 4, animals had a mean RT of 820.9 milliseconds (*SD* = 172.7 milliseconds), while artifacts had a mean RT of 817.6 milliseconds (*SD* = 159.9 milliseconds), *t*(67) = 0.17, *p* = .86, 95% CI [−35, 41], *d_z_* = 0.021, *d_rm_* = 0.02, CL = 51%. A Bayesian repeated measures ANOVA revealed moderate evidence for the null hypothesis for Category, BF_01_ = 7.0, and extreme evidence for the alternative hypothesis for Load, ${\text{BF}}_{01}=9.4\times 10^{-14}$.

#### Experiment 6B

**Figure 13.**
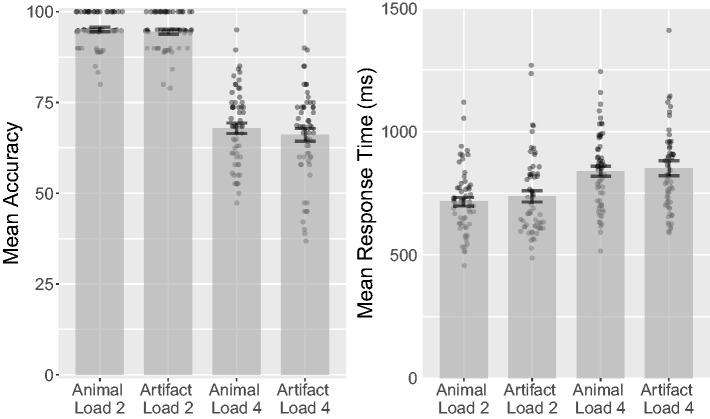
Mean accuracy and RTs in Experiment 6B. Error bars show standard errors.

Before conducting the analysis, we removed six participants for having mean accuracy below 65%. An ANOVA on accuracy over Category (animal, artifact) and Load (Load 2, Load 4) showed a nonsignificant main effect of Category, *F*(1, 57) = 1.38, *p* = .245, $\eta_{p}^{2}$ = .02, $\eta_{g}^{2}$ < .01, but a significant main effect of Load, *F*(1, 57) = 464.36, *p* < .001, $\eta_{p}^{2}$ = .89, $\eta_{g}^{2}$ = .69. The interaction was not significant, *F*(1, 57) = 0.32, *p* = .576, $\eta_{p}^{2}$ < .01, $\eta_{g}^{2}$ < .01. At Load 2, animals had a mean accuracy of 95.1% (*SD* = 4.87%), while artifacts had a mean accuracy of 94.5% (*SD* = 5.17%), *t*(57) = 0.72, *p* = .48, 95% CI [−1.1, 2.3], *d_z_* = 0.094, *d_rm_* = 0.12, CL = 54%. At Load 4, animals had a mean accuracy of 68% (*SD* = 10.9%), while artifacts had a mean accuracy of 66.1% (*SD* = 13.3%), *t*(57) = 0.94, *p* = .35, 95% CI [−2.1, 5.8], *d_z_* = 0.12, *d_rm_* = 0.15, CL = 55% (see Figure 13). A Bayesian repeated measures ANOVA revealed moderate evidence for the null hypothesis for Category, BF_01_ = 6.1, and extreme evidence for the alternate hypothesis for Load, ${\text{BF}}_{01}=2.2\times 10^{-62}$.

Similarly, an ANOVA on RT failed to reach significance for the main effect of Category, *F*(1, 57) = 0.78, *p* = .382, $\eta_{p}^{2}$ = .01, $\eta_{g}^{2}$ < .01, but reached significance for the main effect of Load, *F*(1, 57) = 117.22, *p* < .001, $\eta_{p}^{2}$ = .67, $\eta_{g}^{2}$ = .11. The interaction was also nonsignificant, *F*(1, 57) = 0.07, *p* = .786, $\eta_{p}^{2}$ < .01, $\eta_{g}^{2}$ < .01. At Load 2, animals had a mean RT of 717 milliseconds (*SD* = 132.9 milliseconds), while artifacts had a mean RT of 738 milliseconds (*SD* = 165.7 milliseconds), *t*(57) = 1.6, *p* = .13, 95% CI [−48, 6], *d_z_* = 0.2, *d_rm_* = 0.13, CL = 58%. At Load 4, animals had a mean RT of 839.2 milliseconds (*SD* = 154.1 milliseconds), while artifacts had a mean RT of 851.4 milliseconds (*SD* = 225 milliseconds), *t*(57) = 0.38, *p* = .71, 95% CI [−77, 53], *d_z_* = 0.05, *d_rm_* = 0.063, CL = 52%. A Bayesian repeated measures ANOVA revealed moderate evidence for the null hypothesis for Category, BF_01_ = 4.8, and extreme evidence for the alternate hypothesis for Load, ${\text{BF}}_{01}=3.6\times 10^{-10}$.

#### Additional analysis

To assess the hypothesis that an animal’s increasing size should be particularly pertinent to yield an advantage, we ran an ANOVA on accuracy over Experiment (6A, 6B), Load (Load 1, Load 2), Category (animal, artifact), and Change (larger, smaller). Only the effect of Load reached significance, *F*(1, 124) = 1,008.26, *p* < .001, $\eta_{p}^{2}$ = .89, $\eta_{g}^{2}$ = .51. For brevity, we mention only the most relevant nonsignificant effects here (all other *p*s > .16); effect of Category, *F*(1, 124) = 1.32, *p* = .253, $\eta_{p}^{2}$ = .01, $\eta_{g}^{2}$ < .01, effect of Change, *F*(1, 124) = 0.91, *p* = .341, $\eta_{p}^{2}$ < .01, $\eta_{g}^{2}$ < .01, and interaction between Category and Change, *F*(1, 124) = 1.07, *p* = .304, $\eta_{p}^{2}$ < .01, $\eta_{g}^{2}$ < .01. A Bayesian repeated measures ANOVA showed strong evidence for the null hypothesis of Category, BF_01_ = 11.1 and Change, BF_01_ = 11.8.

Finally, we ran the same ANOVA on RTs. Only the effect of Load reached significance, *F*(1, 119) = 156.77, *p* < .001, $\eta_{p}^{2}$ = .57, $\eta_{g}^{2}$ = .08. None of the other effects were significant; effect of Category, *F*(1, 119) = 0.13, *p* = .715, $\eta_{p}^{2}$ < .01, $\eta_{g}^{2}$ < .01, effect of Change, *F*(1, 119) = 0.56, *p* = .457, $\eta_{p}^{2}$ < .01, $\eta_{g}^{2}$ < .01, and interaction between Category and Change, *F*(1, 119) = 0.13, *p* = .723, $\eta_{p}^{2}$ < .01, $\eta_{g}^{2}$ < .01 (all other *p*s > .13). A Bayesian repeated measures ANOVA showed strong evidence for the null hypothesis of Category, BF_01_ = 12.4, and Change, BF_01_ = 10.3.

### Discussion

None of the observations we had made in Experiment 5 were replicated. In these new experiments, animals were not reported to change significantly more accurately or faster than artifacts in either experiments. This was further confirmed by the obtained BFs which showed moderate evidence for the null hypothesis, supporting the conclusion that there was no effect of category on both accuracy and RTs. Moreover, the combined additional analysis revealed strong evidence for the null hypothesis. It thus appears that specific types of visual transformation can influence how well the stimuli used can be monitored for changes. As Experiments 5A and 6A used the same set of images, the advantage cannot be attributed to a particular set of images being easier to monitor for changes in general. Thus, changes in lateral orientation would seem more relevant for the hypothesized animate monitoring system than changes in size (or depth). Such a conclusion is, however, puzzling, as a change in size would intuitively signal that an animal is either getting closer or further away from the viewer and it would seem that this should be a more salient event (in survival terms) than seeing the same animal changing its direction to the left or right. Indeed, this consideration leads us to think that the results of Experiment 5 might have resulted from some low-level features’ changes that were more noticeable in the lateral orientation of animals. In addition, while it would seem intuitive that animals getting closer should be particularly capable in grabbing attention, we found no support for this. In relation to all, the null results of our previous experiments, as well as the findings with other paradigms, also showing no special role for animals in attention (Hagen & Laeng, 2016, 2017), it seems appropriate to suggest that what we observed in Experiment 5 is more likely to be related to aspects that are not directly related to a general prioritization in attentional processes (e.g., uncontrolled low-level features). Therefore, considering the conflicting results of Experiment 5 in context of the available evidence, we are inclined to conclude that Experiment 5 does not provide strong or conclusive evidence in favor of the animate monitoring mechanism.

An interesting observation from the above experiments is that accuracy appears to be lower in combination with faster RTs as compared with Experiment 5. This could indicate that when changes in size were noticed, this occurred quite rapidly, while changes in lateral orientations appear to require more processing of the visual features for some length of time after the event, as reflected in longer RTs and higher accuracy.

We should note that at present the MEM task is not a time-honored, standard, task in the cognitive sciences, thus not much is known about its limits and caveats. More research is needed to better understand the nature of the task and whether accuracy and RTs are actually indicative of attentional prioritizations of the depicted items. Such research may throw light on whether findings like those of Experiment 5 is likely to be a false positive in relation to studying the proposed innate attentional bias for animal stimuli, or conversely, the present experiment is likely to be a false negative.

## Omnibus Analysis

To provide an overview of all results obtained in this study and make some principled conclusion, we conducted a final omnibus analysis; whereas several of our experiments failed to reach significance or provide conclusive evidence, they could still provide more robust indications of an animal or artifact bias when taken together. Although we based sample sizes on power-based statistical inference, one cannot exclude the possibility that the present experiments could have been underpowered, especially if there is a true effect that is smaller than the effect size originally estimated in the power analysis. Importantly, nonsignificant results are mostly inconclusive when considered alone. Thus, a more stringent way to reach a conclusion is to consider multiple experiments simultaneously, as we attempt to do next.

Although it is statistically impossible to support an effect size of exactly zero (Lakens, 2017), it is possible to use an equivalence test (Lakens, 2017) to reject effect sizes above a specified limit by showing that our estimated effect sizes are statistically smaller than a specified equivalence bound (e.g., [−0.4, 0.4]). However, in absence of a concretely defined theoretical limit for a minimum effect size, we decided to set equivalence bounds to the range of effect sizes that we estimated to have 80% power to find equivalence for (Lakens, 2017) by simulating meta-analyses with heterogeneity similar to our combined experiments.

In addition, to provide some context, we sought to quantify the effect of animal targets relative to another known effect in the field. Specifically, we compared it with the effect of increasing the number of targets (tracking load) in a standard MOT task. Previous work in our lab (Alnæs et al., 2014) has shown that either adding or removing one target leads to an average change in tracking performance of about 5.8%.

To more specifically investigate the effect of category on tracking and monitoring accuracy, we ran an omnibus ANOVA across all experiments, which is similar to running a meta-analysis of raw mean differences (Bond, Wiitala, & Richard, 2003). More specifically, we combined the accuracy measures from Experiment 1 to Experiment 2 and Supplementary Experiments 1 and 3, the position accuracy measures from Experiment 3 to Experiment 4 and Supplementary Experiment 2, and the accuracy measures from Experiment 5 and 6. This omnibus analysis revealed significant main effects of Experiment, *F*(10, 647) = 30.78, *p* < .001, $\eta_{p}^{2}$ = .32, $\eta_{g}^{2}$ = .29 and Category, *F*(1, 647) = 6.13, *p* = .014, $\eta_{p}^{2}$ = .0093, $\eta_{g}^{2}$ = 0.0013. The interaction was also significant, *F*(10, 647) = 1.96, *p* = .036, $\eta_{p}^{2}$ = .03, $\eta_{g}^{2}$ = 0.0042. Equivalence testing on these measures showed that our meta-analytic effect size was not statistically smaller than a *d_z_* of 0.16, *p* = .12. In addition, a Bayesian repeated measures ANOVA revealed only anecdotal and inconclusive evidence for the null hypothesis for Category, BF_01_ = 1.077, and extreme evidence for the alternative hypothesis of Experiment, ${\text{BF}}_{01}=7.05\times 10^{-46}$. Animal targets had a mean accuracy of 78.4% (*SD* = 12.4%), while artifacts had a mean of 77.7% (*SD* = 11.9%). This is an advantage for animal targets of 0.71%, which amounts to a load-relative performance change of 12%, comparable to tracking about 0.12 more objects in a standard MOT task.

Next, we inspected Cook’s distances for the individual effect sizes across experiments and found that measures in Experiments 5A and 5B were likely to have comparatively large impacts on our result. As discussed previously, we have raised doubts on the appropriateness of these experiments, considered in relation to their weak evidential value and conflicting results, the null results of Experiment 6, and the general set of results. Thus, to further assess the weight of Experiment 5 in reaching a significant result in our omnibus analysis, we removed it from the analysis.

Running the same analysis without Experiment 5 showed a significant main effect of Experiment, *F*(8, 537) = 16.10, *p* < .001, $\eta_{p}^{2}$ = .19, $\eta_{g}^{2}$ = .17, but not for Category, *F*(1, 537) = 0.37, *p* = .54, $\eta_{p}^{2}$ = .0007, $\eta_{g}^{2}$ = .00008 or the interaction, *F*(8, 537) = 0.67, *p* = .72, $\eta_{p}^{2}$ = .01, $\eta_{g}^{2}$ = .001. Equivalence testing showed that our meta-analytic effect size was statistically smaller than *d_z_* = 0.145, *p* = .002, indicating that we can reject effect sizes larger than 0.145. A Bayesian repeated measures ANOVA revealed strong evidence for the null hypothesis for Category, BF_01_ = 12.45, and extreme evidence for the alternate hypothesis for Experiment, ${\text{BF}}_{01}=5.05\times 10^{-19}$. Animal targets had a mean accuracy of 76% (*SD* = 11.9%), while artifacts had a mean of 75.8% (*SD* = 11.7%). This is an advantage for animal targets of 0.2%, which is equivalent to tracking about 0.03 more objects in a standard MOT task.

Next, we assessed identity accuracy across Experiments 3 and 4 and Supplementary Experiment 2. This showed significant main effects for Experiment, *F*(2, 187) = 18.67, *p* < .001, $\eta_{p}^{2}$ = .17, $\eta_{g}^{2}$ = .15, and Category, *F*(1, 187) = 6.29, *p* = .013, $\eta_{p}^{2}$ = .03, $\eta_{g}^{2}$ = .003, indicating that animal identities were reported more accurately than artifacts. The interaction was not significant, *F*(2, 187) = 0.24, *p* = .787, $\eta_{p}^{2}$ < .001, $\eta_{g}^{2}$ < .001. Equivalence testing showed that we cannot find statistical support for meta-analytic effect sizes smaller than *d_z_* = 0.234, *p* = .36. A Bayesian repeated measures ANOVA revealed anecdotal evidence for the alternate hypothesis for Category, BF_01_ = 0.419, and extreme evidence for the alternate hypothesis of Experiment, ${\text{BF}}_{01}=3.9\times 10^{-6}$. Animal targets had a mean identity accuracy of 61.5% (*SD* = 14.8%), while artifacts had a mean of 59.9% (*SD* = 14.8%). This is an advantage for animal targets of 1.6%, which can be compared to tracking about 0.27 more objects in a standard MOT task.

When we assessed the same experiments for position accuracy, we found a significant main effect of Experiment, *F*(2, 187) = 16.22, *p* < .001, $\eta_{p}^{2}$ = .15, $\eta_{g}^{2}$ = .13, but no significant effect of Category, *F*(1, 187) = 0.03, *p* = .866, $\eta_{p}^{2}$ = 0.00015, $\eta_{g}^{2}$ < .0001, neither was the interaction significant, *F*(2, 187) = 1.05, *p* = .351, $\eta_{p}^{2}$ = .01, $\eta_{g}^{2}$ < .001. Equivalence testing showed that we can reject meta-analytic effect sizes larger than *d_z_* = 0.273, *p* = .03. A Bayesian repeated measures ANOVA revealed moderate evidence for the null hypothesis for Category, BF_01_ = 8.96, and evidence for the alternate hypothesis for Experiment, ${\text{BF}}_{01}=2.6\times 10^{-5}$. Animal targets had mean position accuracy of 74.5% (*SD* = 13.1%), while artifacts had a mean of 74.4% (*SD* = 12.5%). This is an advantage for animal targets of 0.1%, which is equivalent to tracking about 0.02 more objects in a standard MOT task.

We also ran an omnibus ANOVA on percentages of incorrect responses in Experiments 1 to 4 and Supplementary Experiments 1, 2, and 3. This analysis showed nonsignificant main effects of Experiment, *F*(6, 408) < 0.001, *p* > .999, $\eta_{p}^{2}$ < .001, $\eta_{g}^{2}$ < .001, and Category, *F*(1, 408) = 0.06, *p* = .80, $\eta_{p}^{2}$ < .001, $\eta_{g}^{2}$ < .001. The interaction was also nonsignificant, *F*(6, 408) = 0.38, *p* = .89, $\eta_{p}^{2}$ < .01, $\eta_{g}^{2}$ < .01. Equivalence testing on this measure showed that we can reject meta-analytic effect sizes larger than *d_z_* = 0.149, *p* = .003. A Bayesian repeated measures ANOVA revealed strong evidence for the null hypothesis for Category, BF_01_ = 11.1, and Experiment, BF_01_ = 2,592.3.

Finally, we conducted an omnibus ANOVA on response orders across Experiments 1 to 2 and Supplementary Experiments 1 and 3. This analysis revealed a significant main effect of Experiment, *F*(3, 226) = 651.2, *p* < .001, $\eta_{p}^{2}$ = .90, $\eta_{g}^{2}$ = .87, and a nonsignificant main effect of Category, *F*(1, 226) = 1.35, *p* = .25, $\eta_{p}^{2}$ = .006, $\eta_{g}^{2}$ = .002. The interaction was also not significant, *F*(3, 226) = 0.46, *p* = .71, $\eta_{p}^{2}$ = .006, $\eta_{g}^{2}$ = .002. Equivalence testing showed that we can reject meta-analytic effect sizes larger than *d_z_* = 0.20, *p* = .02. A Bayesian repeated measures ANOVA revealed moderate evidence for the null hypothesis for Category, BF_01_ = 4.8, and extreme evidence for the alternate hypothesis for Experiment, ${\text{BF}}_{01}=3.9\times 10^{-108}$.

In more concrete terms, ignoring averages per participant, we obtained 107,374 valid responses from 658 participants across all experiments (e.g., excluding responses before a change occurred in the MEM tasks). Of these, 40,620 were correctly reported animals and 40,457 were correctly reported artifacts (ignoring identity accuracies). This amounts to a difference in only 163 responses. Thus, across 81,077 human attentional engagements with animal stimuli, just 0.2% of them represent our overall observed bias in tracking and monitoring accuracy (or 0.67% by exchanging position accuracy for identity accuracy). A similar count on incorrect responses revealed that 11,790 animal distractors and 11,789 artifact distractors were incorrectly reported as targets, a difference of only one response.

Based on the above set of analyses, we cannot conclude that animals had a sizable influence on performance across tasks. Although we cannot entirely conclude that animals had no effect across tasks either, it turned out that in a majority of our tests, we got moderate to strong evidence for no effect of animals, while only inconclusive evidence for an effect in omnibus analyses that involved Experiment 5 and identity accuracy. Hence, it seems difficult to interpret these results as robustly supporting the animate monitoring hypothesis. All in all, we found evidence for no effect of images of animals in omnibus ANOVAs on tracking and monitoring accuracy (depending on the exclusion of Experiment 5), percentages of incorrect responses, and response orders. In fact, we found support for effect sizes smaller than what we had 80% power to find equivalence for. However, our data were not sensitive enough to conclude whether animals had an influence on identity accuracy or not. If the indicated effect sizes are deemed interesting and worthwhile, future studies should aim to include more participants than in this study as well as reassessing the validity of the design of Experiment 5.

## General Discussion

On the basis of the animate monitoring hypothesis (New et al., 2007), we have used several versions of the MOT, MIT, and MEM tasks to investigate (a) whether images of animals can improve position and identity tracking, (b) whether they can act as more effective distractors, (c) whether they are selected prior to artifacts in the response phase, and (d) whether they are easier to monitor for changes. In the first three experiments with MOT and MIT tasks, we failed to reject the null hypothesis of no advantage or bias for animal targets and distractors. In fact, we found evidence in support of the null hypothesis. In Experiment 4, however, we did uncover a significant advantage for animal targets in identity accuracy but not in position accuracy. Remarkably, we observed the opposite: a significant advantage for artifacts. A Bayesian test did, however, show that the evidence for such effects was only weak and inconclusive. In the following two experiments (5A, 5B), instructing participants to monitor for lateral orientation changes, seemingly uncovered a pattern of results consistent with the animate monitoring hypothesis. As animals were reported significantly more accurately than artifacts in both experiments, and animal targets were reported significantly faster than artifacts in Experiment 5A. However, a Bayesian test did not support such a conclusion, as the evidence for an effect of category on accuracy turned out to only be anecdotal in both experiments. The effect of category on RT was also conflicting, as the first experiment (5A) showed moderate evidence for the alternate hypothesis while the latter (5B) showed moderate evidence for the null hypothesis. To follow up on these observations and to attempt to rule out that the indications of an advantage was specific to the type of change used, we substituted the lateral orientation changes with changes in size (as an index of proximity). This substitution appeared to be crucial for an advantage for animals in accuracy and RTs, as we consistently found moderate evidence for the null hypothesis of no effect of category. In fact, this pattern of results lends credibility to the interpretation that the results of Experiment 5 could represent a potential false positive in relation to an attentional bias for animals. More specifically, given the fact that Experiment 6A used the same set of images as Experiment 5A and failed to replicate any bias, this may support the interpretation that the specific task in combination with the image set could have had effects that were actually independent of a specific attentional bias to animals (e.g., low-level features being more salient in lateral views of animals than artifacts in general). While some of our results could potentially be viewed as false negatives or, at least, as not sensitive enough to reveal the supposed attentional bias, it seems that if a true effect of images of animals on our measures exists at all, it is likely to be small.

Based on our omnibus approach, we failed to obtain robust evidence for appreciable effect sizes across measures for position accuracy, incorrect responses (distractibility), response order (response prioritization), as well as monitoring RTs and accuracy. We did, however, find indications of improved identity accuracy for animal targets, but this effect was difficult to consider as evidence for an attentional bias given the overall context of the results. In fact, our degree of evidence in support of the presence of an effect for animals is largely dependent on the inclusion of Experiment 5, thus it seems important that future studies assess the validity of this experimental design in studying attentional biases. In fact, we can reject small-to-medium effect sizes with equivalence bounds when disregarding Experiment 5 in an omnibus analysis. Similarly, we found support for rejecting effect sizes considerably smaller than what we expected to find for measures on location accuracy, incorrect responses, and response orders. In the end, it cannot be ruled out that if there is a true effect, it might be smaller than our equivalence bounds and what our study was powered for. Hence, studies with a considerably higher level of statistical power should be conducted in future investigations, assuming that the potential for such small effects is deemed interesting and worthwhile.

We need to point out that we have attempted to tax and challenge the attentional system responsible for keeping track of objects in various ways to bring about errors such that a category-specific prioritization may become detectable. However, it seems that a robust advantage or distracting effect of animals is not easily obtained in tasks requiring divided sustained attention. Rather, it seems that special conditions may need to be met to detect a sizable advantage, which raises the question of the validity and aptness of those conditions in revealing an attentional bias.

In fact, the observed effect sizes across experiments were considerably smaller than what the authors of the original hypothesis designed for in their seminal study (New et al., 2007). Clearly, we cannot entirely rule out the presence of a small effect of animal targets. One should, however, consider the extent in which such a weak effect has practical implications for survival as well as the validity of the measures used to obtain such an effect. In particular, it is difficult to envision that lateral orientation changes should be especially sensitive to an attentional bias for animals in relation to the other null results and the original formulation of the hypothesis. In fact, it would seem necessary to reformulate the hypothesis, in order to provide a reason for why the attentional bias should be more sensitive to changes in lateral orientation of animals, without significantly improving tracking performance, or biasing response orders, or allowing them to act as more effective distractors than artifacts. For these reasons, it seems to us difficult to argue that an evolved animal monitoring circuit should be especially sensitive to changes in lateral orientation instead of the size (or proximity) of an animal. In fact, it does not seem particularly adaptive for a putative monitoring system to be specifically sensitive to some features that do not explicitly appear as more relevant for survival than others. Moreover, we cannot assume that performance in the MEM task with lateral orientation changes can be seen as exclusively grounded in attentional ability or attentional processes. There is the possibility that an advantage in detecting the type of changes made to animals over artifacts is based on cues provided by low-level features that were present in the sets of stimuli and not due to their category per se. However, we cannot rule out that sensitivity to specific low-level features could have been selected itself by natural selection to assist the detection of a specific category of objects (e.g., eye-like shapes. suggesting different directions of movement).

In addition, it seems highly relevant to judge the present evidence in the light of other experiments and experimental paradigms that have examined the prioritization of animal stimuli in attention. In particular, in our laboratory, we have previously used tasks such as change detection (Hagen & Laeng, 2016) and attentional blink (Hagen & Laeng, 2017), which have both led us to question the animate monitoring hypothesis in relation to prioritization of attentional mechanisms. Other researchers have employed inattentional blindness tasks, which apparently showed an advantage for animals (Calvillo & Hawkins, 2016; Calvillo & Jackson, 2014). However, our previous study with images of animals in an attentional blink task showed that animals have no considerable impact on attentional blinks (Hagen & Laeng, 2017), but that they are reported more successfully regardless of their temporal position, suggesting instead an advantage in perceptual processing or encoding. That is, animal stimuli are unable to surpass the blindness of the attentional blink or spontaneously induce such blinks, while other stimuli considered biologically important (e.g., arousing words, facial expressions, or food) do seem to be able to do this.

We note that another line of work (Pratt et al., 2010) has investigated the ability of arbitrarily shaped objects (squares) to capture attention when they abruptly changed motion patterns from predictable trajectories to unpredictable animate trajectories. The changes were coupled with a type of change detection task, where participants reported a vanishing square shortly after the change in motion pattern occurred. The experiments quite successfully demonstrated that such animate motion patterns are able to capture attention. An interesting question in relation to this study is whether such changes in motion patterns would be more readily detected if the objects were depicted as animals as opposed to artifacts. It is also worth noting that this study used random motion trajectories, which according to Pratt et al. (2010), should signal animate motion, as animates rarely move in predictable or lawful ways (they are self-propelled rather than moving under Newtonian physics). While our designs are markedly different from that of Pratt et al. (2010), one may raise the concern that the random motion patterns employed in the present experiments could have signaled animacy for the animate monitoring system. Hence, participants could have assigned equal priority to all objects, irrespective of what they depicted, effectively erasing any potential for a bias for images of animals. To attempt to resolve this concern, we have included Supplementary Experiment 3, which found no discernible evidence for a bias toward objects moving with random and unpredictable directional changes as compared with objects moving predictably. Importantly, it did not show that images of animals were biased when moving with predictable motions. In fact, the evidence was markedly incompatible with the aforementioned concern.

Another potential concern with the design of the present experiments is that the system responsible for assigning attentional biases to animals could have been overtaxed by the number of animals displayed simultaneously. However, considering that research employing a large number of facial stimuli in similar tasks have repeatedly been successful in showing attentional biases (Jin & Xu, 2015; Li et al., 2016, 2017; Liu & Chen, 2012), this does not appear as a significant concern, and even less so regarding our last two experiments. Future studies could, however, attempt, with appropriate balancing, trials with just one animal among multiple nonanimals to discern whether it will be prioritized more than a single nonanimal or multiple animals.

Finally, we would also like to note that, in contrast to our individual controlled experiments, the actual natural environment poses a complex and varied set of challenges. The present experiments represent only a modest probe in comparison to a noisy environment where animal monitoring may be relevant under different circumstances. Thus, researchers should pose the question of whether attentional biases for animals are even measurable in laboratory settings. Perhaps using a computer monitor showing images of animals cannot bring about robust evidence for an attentional bias, as these are confined to the use of *symbolic* representations, not the natural stimuli themselves. It is possible that our perceptual and attentional systems are perfectly able to make a clear distinction between image-like representations or depictions and the real objects (e.g., a deer vs. a car entering your visual field). Perhaps only the natural stimuli are fully able to engage perceptual or cognitive mechanisms in a manner consistent with the theory. However, we deem this possibility unlikely, as most of the evidence in favor of evolutionary processes on perception (e.g., properties of faces) and on attention (e.g., pop-out features) have been based on laboratory demonstrations with flattened, often abstract, and symbolic depictive items. Nevertheless, our point is that drawing conclusions from controlled settings to naturalistic behavior should be done with considerable caution and research in more naturalistic settings should be considered for future research, before attempting to conclude on how humans behave in the natural world when encountering other animals.

## Conclusion

We have studied the role of animals in attention by challenging over 600 participants with several variations of visual tracking tasks, all requiring divided and sustained attention. Following the reasoning behind the animate monitoring hypothesis, we expected to find that associating positions with images of animals would lead to more accurate tracking, more vigilant monitoring, prioritized responses, and that animals would function as more effective distractors than artifacts. The combined results are, however, not strongly or unequivocally supportive of these expectations. Although some observations were in favor of the animate monitoring hypothesis, these could not be regarded as more than weak and inconclusive evidence. Indeed, the indicated effect sizes across experiments were considerably smaller than what we expected to find from current theory and previous research. We found moderate to strong evidence that images of animals do not improve positional tracking, do not act as more effective distractors, are not selected prior to artifacts in the response phase, and are not easier to monitor for changes in size.

## Supplemental Material

Supplemental material for Chasing Animals With Split Attention: Are Animals Prioritized in Visual Tracking? Click here for additional data file. Supplemental material for Chasing Animals With Split Attention: Are Animals Prioritized in Visual Tracking? by Thomas Hagen, Thomas Espeseth and Bruno Laeng in i-Perception

## References

[bibr1-2041669518795932] Alnæs D. Sneve M. H. Espeseth T. Endestad T. van de Pavert S. H. P. Laeng B. (2014) Pupil size signals mental effort deployed during multiple object tracking and predicts brain activity in the dorsal attention network and the locus coeruleus. Journal of Vision 14: 1–20. 10.1167/14.4.1 24692319

[bibr2-2041669518795932] Andraszewicz S. Scheibehenne B. Rieskamp J. Grasman R. Verhagen J. Wagenmakers E.-J. (2015) An introduction to Bayesian hypothesis testing for management research. Journal of Management 41: 521–543.

[bibr63-2041669518795932] Altman, M. N., Khislavsky, A. L., Coverdale, M. E., & Gilger, J. W. (2016). Adaptive attention: how preference for animacy impacts change detection. *Evolution and Human Behavior*, *37*(4), 303–314.

[bibr3-2041669518795932] Bahrami B. (2003) Object property encoding and change blindness in multiple object tracking. Visual Cognition 10: 949–963.

[bibr4-2041669518795932] Bates E. D’Amico S. Jacobsen T. Székely A. Andonova E. Devescovi A. Wicha N. (2003) Timed picture naming in seven languages. Psychonomic Bulletin & Review 10: 344–380. 12921412 10.3758/bf03196494 PMC3392189

[bibr62-2041669518795932] Bond Jr, C. F., Wiitala, W. L., & Richard, F. D. (2003). Meta-analysis of raw mean differences. *Psychological Methods*, *8*(4), 406–418. 10.1037/1082-989X.8.4.406 14664679

[bibr5-2041669518795932] Bonin P. Peereman R. Malardier N. Méot A. Chalard M. (2003) A new set of 299 pictures for psycholinguistic studies: French norms for name agreement, image agreement, conceptual familiarity, visual complexity, image variability, age of acquisition, and naming latencies. Behavior Research Methods, Instruments, & Computers 35: 158–167. 10.3758/bf03195507 12723790

[bibr6-2041669518795932] Calvillo D. P. Hawkins W. C. (2016) Animate objects are detected more frequently than inanimate objects in inattentional blindness tasks independently of threat. The Journal of General Psychology 143: 101–115. 27055078 10.1080/00221309.2016.1163249

[bibr7-2041669518795932] Calvillo D. P. Jackson R. E. (2014) Animacy, perceptual load, and inattentional blindness. Psychonomic Bulletin & Review 21: 670–675. 24197657 10.3758/s13423-013-0543-8

[bibr8-2041669518795932] Capitani E. Laiacona M. Barbarotto R. Trivelli C. (1994) Living and non-living categories. Is there a normal asymmetry? Neuropsychologia 32: 1453–1463. 7885575 10.1016/0028-3932(94)90117-1

[bibr9-2041669518795932] Capitani E. Laiacona M. Mahon B. Caramazza A. (2003) What are the facts of semantic category-specific deficits? A critical review of the clinical evidence. Cognitive Neuropsychology 20: 213–261. 20957571 10.1080/02643290244000266

[bibr10-2041669518795932] Caramazza A. Shelton J. R. (1998) Domain-specific knowledge systems in the brain: The animate-inanimate distinction. Journal of Cognitive Neuroscience 10: 1–34. 9526080 10.1162/089892998563752

[bibr11-2041669518795932] Cavanagh P. Alvarez G. A. (2005) Tracking multiple targets with multifocal attention. Trends in Cognitive Sciences 9: 349–354. 15953754 10.1016/j.tics.2005.05.009

[bibr12-2041669518795932] Cohen M. A. Pinto Y. Howe P. D. Horowitz T. S. (2011) The what–where trade-off in multiple-identity tracking. Attention, Perception, & Psychophysics 73: 1422–1434. 10.3758/s13414-011-0089-7 21380611

[bibr13-2041669518795932] Crump M. J. McDonnell J. V. Gureckis T. M. (2013) Evaluating Amazon’s Mechanical Turk as a tool for experimental behavioral research. PLoS One 8: e57410. 23516406 10.1371/journal.pone.0057410 PMC3596391

[bibr14-2041669518795932] Cycowicz Y. M. Friedman D. Rothstein M. Snodgrass J. G. (1997) Picture naming by young children: Norms for name agreement, familiarity, and visual complexity. Journal of Experimental Child Psychology 65: 171–237. 9169209 10.1006/jecp.1996.2356

[bibr15-2041669518795932] Dienes Z. (2014) Using Bayes to get the most out of non-significant results. Frontiers in Psychology 5: 1–17. 25120503 10.3389/fpsyg.2014.00781 PMC4114196

[bibr16-2041669518795932] Drew T. Horowitz T. S. Vogel E. K. (2013) Swapping or dropping? Electrophysiological measures of difficulty during multiple object tracking. Cognition 126: 213–223. 23141025 10.1016/j.cognition.2012.10.003 PMC3529852

[bibr17-2041669518795932] Gainotti G. (2000) What the locus of brain lesion tells us about the nature of the cognitive defect underlying category-specific disorders: A review. Cortex 36: 539–559. 11059454 10.1016/s0010-9452(08)70537-9

[bibr18-2041669518795932] Gainotti G. (2010) The influence of anatomical locus of lesion and of gender-related familiarity factors in category-specific semantic disorders for animals, fruits and vegetables: A review of single-case studies. Cortex 46: 1072–1087. 20472230 10.1016/j.cortex.2010.04.002

[bibr19-2041669518795932] Gainotti G. (2015) Inborn and experience-dependent models of categorical brain organization. A position paper. Frontiers in Human Neuroscience 9: 1–19. 25667570 10.3389/fnhum.2015.00002 PMC4304236

[bibr20-2041669518795932] Hagen T. Laeng B. (2016) The change detection advantage for animals: An effect of ancestral priorities or progeny of experimental design? i-Perception 7((3): 1–17. doi: 10.1177/2041669516651366. 10.1177/2041669516651366 PMC4934668 27433331

[bibr21-2041669518795932] Hagen T. Laeng B. (2017) Animals do not induce or reduce attentional blinking, but they are reported more accurately in a rapid serial visual presentation task. i-Perception 8((5): 1–25. doi: 10.1177/2041669517735542. 10.1177/2041669517735542 PMC5648101 29085619

[bibr22-2041669518795932] Hillis A. E. Caramazza A. (1991) Category-specific naming and comprehension impairment: A double dissociation. Brain 114: 2081–2094. 1933235 10.1093/brain/114.5.2081

[bibr23-2041669518795932] Horowitz T. S. Klieger S. B. Fencsik D. E. Yang K. K. Alvarez G. A. Wolfe J. M. (2007) Tracking unique objects. Attention, Perception, & Psychophysics 69: 172–184. 17557588 10.3758/bf03193740

[bibr24-2041669518795932] Jackson R. E. Calvillo D. P. (2013) Evolutionary relevance facilitates visual information processing. Evolutionary Psychology 11: 1011–1026. 24184882 10.1177/147470491301100506 PMC10437104

[bibr25-2041669518795932] Jarosz A. F. Wiley J. (2014) What are the odds? A practical guide to computing and reporting Bayes factors. The Journal of Problem Solving 7: 2–9.

[bibr27-2041669518795932] Jin H. Xu B. (2015) The effect of fearful expressions on multiple face tracking. Psychologica Belgica 55: 101–117. 10.5334/pb.bi PMC5854165 30479419

[bibr28-2041669518795932] Kahneman D. (1973) Attention and effort, Englewood Cliffs, NJ: Prentice Hall.

[bibr29-2041669518795932] Konkle T. Caramazza A. (2013) Tripartite organization of the ventral stream by animacy and object size. Journal of Neuroscience 33: 10235–10242. 23785139 10.1523/JNEUROSCI.0983-13.2013 PMC3755177

[bibr30-2041669518795932] Kriegeskorte N. Mur M. Ruff D. A. Kiani R. Bodurka J. Esteky H. Bandettini P. A. (2008) Matching categorical object representations in inferior temporal cortex of man and monkey. Neuron 60: 1126–1141. 19109916 10.1016/j.neuron.2008.10.043 PMC3143574

[bibr31-2041669518795932] Låg T. (2005) Category-specific effects in object identification: What is normal? Cortex 41: 833–841. 16350663 10.1016/s0010-9452(08)70302-2

[bibr32-2041669518795932] Låg T. Hveem K. Ruud K. P. Laeng B. (2006) The visual basis of category effects in object identification: Evidence from the visual hemifield paradigm. Brain and Cognition 60: 1–10. 16182424 10.1016/j.bandc.2005.08.002

[bibr33-2041669518795932] Lakens D. (2013) Calculating and reporting effect sizes to facilitate cumulative science: A practical primer for t-tests and ANOVAs. Frontiers in Psychology 4: 1–12. 24324449 10.3389/fpsyg.2013.00863 PMC3840331

[bibr34-2041669518795932] Lakens D. (2017) Equivalence tests: A practical primer for t tests, correlations, and meta-analyses. Social Psychological and Personality Science 8: 355–362. 28736600 10.1177/1948550617697177 PMC5502906

[bibr35-2041669518795932] Laws K. R. Hunter M. Z. (2006) The impact of colour, spatial resolution, and presentation speed on category naming. Brain and Cognition 62: 89–97. 16938371 10.1016/j.bandc.2006.03.002

[bibr36-2041669518795932] Laws K. R. Neve C. (1999) A ‘normal’ category-specific advantage for naming living things. Neuropsychologia 37: 1263–1269. 10530726 10.1016/s0028-3932(99)00018-4

[bibr37-2041669518795932] Li J. Oksama L. Hyönä J. (2016) How facial attractiveness affects sustained attention. Scandinavian Journal of Psychology 57: 383–392. 27347672 10.1111/sjop.12304

[bibr38-2041669518795932] Li J. Oksama L. Nummenmaa L. Hyönä J. (2018) Angry faces are tracked more easily than neutral faces during multiple identity tracking. Cognition and Emotion 32: 464–479. 28402215 10.1080/02699931.2017.1315929

[bibr39-2041669518795932] Liu C. H. Chen W. (2012) Beauty is better pursued: Effects of attractiveness in multiple-face tracking. The Quarterly Journal of Experimental Psychology 65: 553–564. 22117090 10.1080/17470218.2011.624186

[bibr40-2041669518795932] Mahon B. Z. Anzellotti S. Schwarzbach J. Zampini M. Caramazza A. (2009) Category-specific organization in the human brain does not require visual experience. Neuron 63: 397–405. 19679078 10.1016/j.neuron.2009.07.012 PMC2743253

[bibr41-2041669518795932] Mahon B. Z. Caramazza A. (2009) Concepts and categories: A cognitive neuropsychological perspective. Annual Review of Psychology 60: 27–51. 10.1146/annurev.psych.60.110707.163532 PMC2908258 18767921

[bibr42-2041669518795932] Nairne J. S. VanArsdall J. E. Cogdill M. (2017) Remembering the living: Episodic memory is tuned to animacy. Current Directions in Psychological Science 26: 22–27.

[bibr43-2041669518795932] Nairne J. S. VanArsdall J. E. Pandeirada J. N. Cogdill M. LeBreton J. M. (2013) Adaptive memory: The mnemonic value of animacy. Psychological Science 24: 2099–2105. 23921770 10.1177/0956797613480803

[bibr44-2041669518795932] New, J., Cosmides, L., & Tooby, J. (2007). Category-specific attention for animals reflects ancestral priorities, not expertise. *Proceedings of the National Academy of Sciences, 104*, 16598–16603. 10.1073/pnas.0703913104 PMC2034212 17909181

[bibr45-2041669518795932] Nishimoto T. Miyawaki K. Ueda T. Une Y. Takahashi M. (2005) Japanese normative set of 359 pictures. Behavior Research Methods 37: 398–416. 16405135 10.3758/bf03192709

[bibr46-2041669518795932] Nummenmaa L. Oksama L. Glerean E. Hyönä J. (2017) Cortical circuit for binding object identity and location during multiple-object tracking. Cerebral Cortex 27: 162–172. 27913430 10.1093/cercor/bhw380 PMC5939196

[bibr47-2041669518795932] Ogawa H. Watanabe K. Yagi A. (2009) Contextual cueing in multiple object tracking. Visual Cognition 17: 1244–1258.

[bibr48-2041669518795932] Oksama L. Hyönä J. (2008) Dynamic binding of identity and location information: A serial model of multiple identity tracking. Cognitive Psychology 56: 237–283. 17451667 10.1016/j.cogpsych.2007.03.001

[bibr49-2041669518795932] Oksama L. Hyönä J. (2016) Position tracking and identity tracking are separate systems: Evidence from eye movements. Cognition 146: 393–409. 26529194 10.1016/j.cognition.2015.10.016

[bibr50-2041669518795932] Op de Beeck H. Wagemans J. (2001) Visual object categorisation at distinct levels of abstraction: A new stimulus set. Perception 30: 1337–1361. 11768488 10.1068/p3120

[bibr51-2041669518795932] Pinto Y. Howe P. D. Cohen M. A. Horowitz T. S. (2010) The more often you see an object, the easier it becomes to track it. Journal of Vision 10: 4–4. 10.1167/10.10.4 PMC2951308 20884469

[bibr52-2041669518795932] Pratt J. Radulescu P. V. Guo R. M. Abrams R. A. (2010) Its alive! Animate motion captures visual attention. Psychological Science 21: 1724–1730. 20974713 10.1177/0956797610387440

[bibr53-2041669518795932] Pylyshyn Z. (2004) Some puzzling findings in multiple object tracking: I. Tracking without keeping track of object identities. Visual Cognition 11: 801–822.

[bibr54-2041669518795932] Schiff W. Caviness J. A. Gibson J. J. (1962) Persistent fear responses in rhesus monkeys to the optical stimulus of “looming.”. Science 136: 982–983. 14498362 10.1126/science.136.3520.982

[bibr55-2041669518795932] Sha, L., Haxby, J. V., Abdi, H., Guntupalli, J. S., Oosterhof, N. N., Halchenko, Y. O., & Connolly, A. C. (2015). The animacy continuum in the human ventral vision pathway. *Journal of Cognitive Neuroscience*, *27*, 665–678. 10.1162/jocn_a_00733 25269114

[bibr56-2041669518795932] Simons D. J. Levin D. T. (1997) Change blindness. Trends in Cognitive Sciences 1: 261–267. 21223921 10.1016/S1364-6613(97)01080-2

[bibr57-2041669518795932] Snodgrass J. G. Vanderwart M. (1980) A standardized set of 260 pictures: Norms for name agreement, image agreement, familiarity, and visual complexity. Journal of Experimental Psychology: Human Learning and Memory 6: 174–215. 7373248 10.1037//0278-7393.6.2.174

[bibr58-2041669518795932] Warrington E. K. Shallice T. (1984) Category specific semantic impairments. Brain 107: 829–853. 6206910 10.1093/brain/107.3.829

[bibr59-2041669518795932] Wetzels R. Ravenzwaaij D. Wagenmakers E. (2015) Bayesian analysis. In: Cautin R. L. Lilienfeld S. O. (eds) The encyclopedia of clinical psychology, Malden, MA: John Wiley, pp. 1–11.

[bibr60-2041669518795932] Wiggett A. J. Pritchard I. C. Downing P. E. (2009) Animate and inanimate objects in human visual cortex: Evidence for task-independent category effects. Neuropsychologia 47: 3111–3117. 19631673 10.1016/j.neuropsychologia.2009.07.008

[bibr61-2041669518795932] Wu C.-C. Wolfe J. M. (2016) Multiple event monitoring. Cognitive Research: Principles and Implications 1: 1–12. 28180172 10.1186/s41235-016-0022-7 PMC5256474

