# Supplementary material for: Chasing Animals With Split Attention: Are Animals Prioritized in Visual Tracking?
Source: Iperception. 2018 Sep 3;9(5):2041669518795932. doi: 10.1177/2041669518795932 (PMC6124190; doi:10.1177/2041669518795932)
Supplement: Supplemental material for Chasing Animals With Split Attention: Are Animals Prioritized in Visual Tracking? [file Supplemental_material.pdf]

Chasing animals with split attention: Are animals prioritized in visual tracking?

Supplementary material.

Thomas Hagen, Thomas Espeseth and Bruno Laeng

Department of Psychology, University of Oslo, Oslo, Norway

Chasing animals with split attention: Are animals prioritized in visual tracking?

Supplementary material.

Supplementary Experiment 1

The following experiment is mostly identical to Experiment 2 in the main manuscript, except that the objects were hidden during the tracking phase (see Figure 1).

We hypothesized that targets presented as animals during the target assignment phase would be tracked more successfully. As previously, we also predicted that animal targets should be reported before (clicked on) artifact targets, due to the presumed prioritization process and that animal distractors should be reported as targets more frequently than artifact distractors.

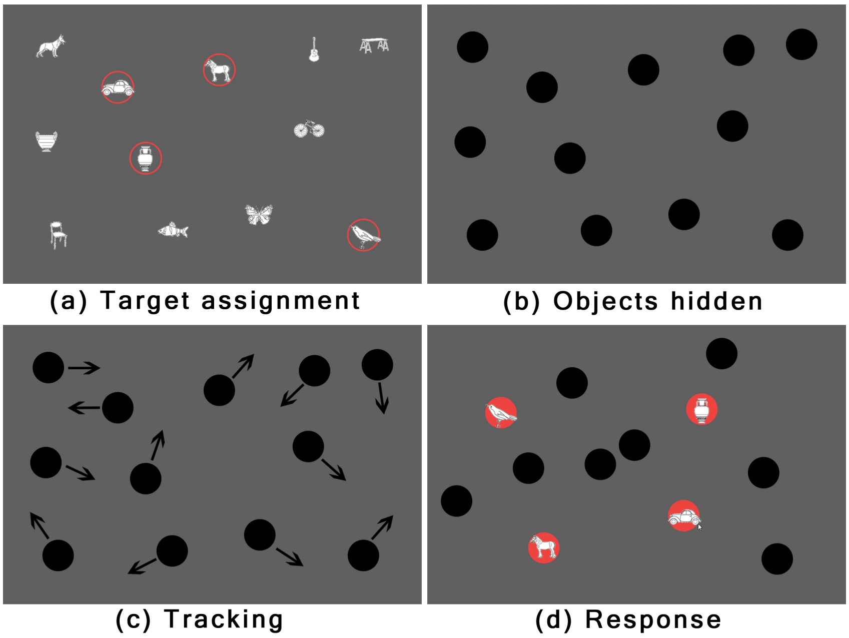

Figure 1. Illustration of a trial in Supplementary Experiment 1. First targets were assigned by enclosing them in red circles (a), then all objects were hidden behind black disks (b) before they started moving around the display (c). Participants indicated the positions of the targets when the movement stopped by clicking on them (d), which made the objects visible.

Methods

Participants. We recruited 59 participants (14 females) with a mean age of 30.2 years (range: 17–49 years, *SD*: 8.34 years). All were recruited in the same way as the previous experiment.

Apparatus. Identical to the other experiments in the main manuscript.

Stimuli. We used the same set of object images as in Experiment 2 in the main manuscript.

Procedure. Identical to Experiment 2 in the main manuscript, except that the objects were hidden behind black disks during the tracking phase and the speed was lowered to 12 pixels per frame to avoid making the task too difficult (see Figure 1).

Results

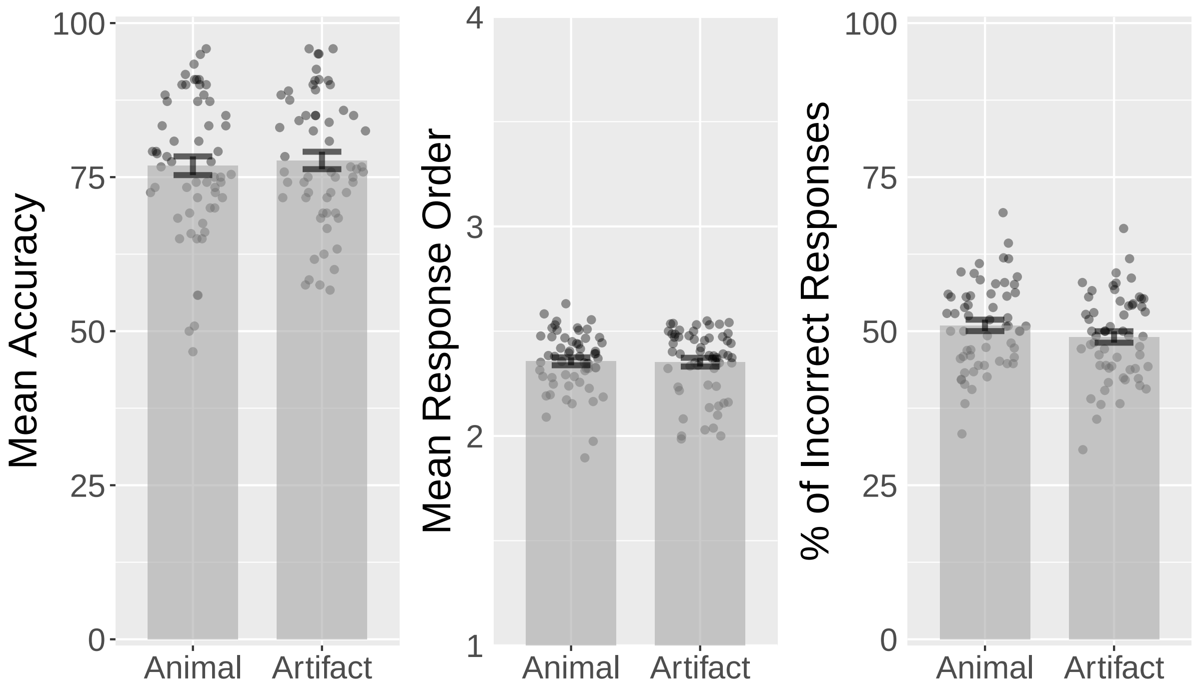

Figure 2. Combined bar and scatter plots on mean accuracy, response orders and percentage of incorrect responses over target category in Supplementary Experiment 1. Error bars show standard errors and the superimposed scatterplots show mean values of each participant.

Before performing statistical analysis we removed 1 participant for having a mean accuracy of less than 50% (1.5 *SD* below the median). A *t*-test on accuracy between animals (M = 76.8%, SD = 11.6%) and artifacts (M = 77.7%, SD = 10.8%) showed no significant difference, $t(57) = 1.5$, $p = 0.14$, 95% *CI* [-2, 0.28], $d_z = 0.2$, $d_{rm} = 0.076$, CL = 58%. A Bayesian t-test showed anecdotal evidence for the null hypothesis, BF_{01} = 2.375.

A *t*-test on mean response order between animals ($M = 2.36$, $SD = 0.14$) and artifacts ($M = 2.35$, $SD = 0.16$) showed no significant difference, $t(57) = 0.24$, p = 0.81, 95% *CI* [-0.029, 0.038], $d_z = 0.032$, $d_{rm} = 0.027$, CL = 51% (see Figure 2). A Bayesian t-test showed moderate evidence for the null hypothesis, BF_{01} = 6.773.

Next we analyzed percentage of incorrect responses between animal (M = 50.9%, SD = 7.1%) and artifact (M = 49.1%, SD = 7.1%) distractors, but found no significant difference, $t(57) = 1$, $p = 0.31$, 95% *CI* [-1.8, 5.6], $d_z = 0.13$, $d_{rm} = 0.27$, CL = 55%. A Bayesian t-test showed moderate evidence for the null hypothesis, BF_{01} = 4.279.

Discussion

Similar to Experiment 2 in the main manuscript we found no significant results for more successful tracking or earlier reporting of targets presented as animals during the target assignment phase. Neither did we find any significant indications of a bias towards animal distractors. The Bayesian t-tests revealed evidence in the same direction as these tests. However, the Bayes factor in favor of the null hypothesis for the effect of category on accuracy was only anecdotal. Thus one should aim to increase the sample size to be able to more conclusively state that there is likely to be no effect.

Supplementary Experiment 2

In an attempt to make the binding of identity and position more volatile and to investigate if associating a moving position with an animal can improve tracking performance, we chose again to hide the objects' identities during the tracking phase.

Piloting of the task showed that tracking the identities of 4 hidden objects appeared to yield an unsuitable level of difficulty, we thus decided to use 3 targets.

Similar to our previous experiments we expected higher accuracy for both identity and position measures for animal targets as compared to artifact targets as well as more frequent reporting of animal distractors as targets.

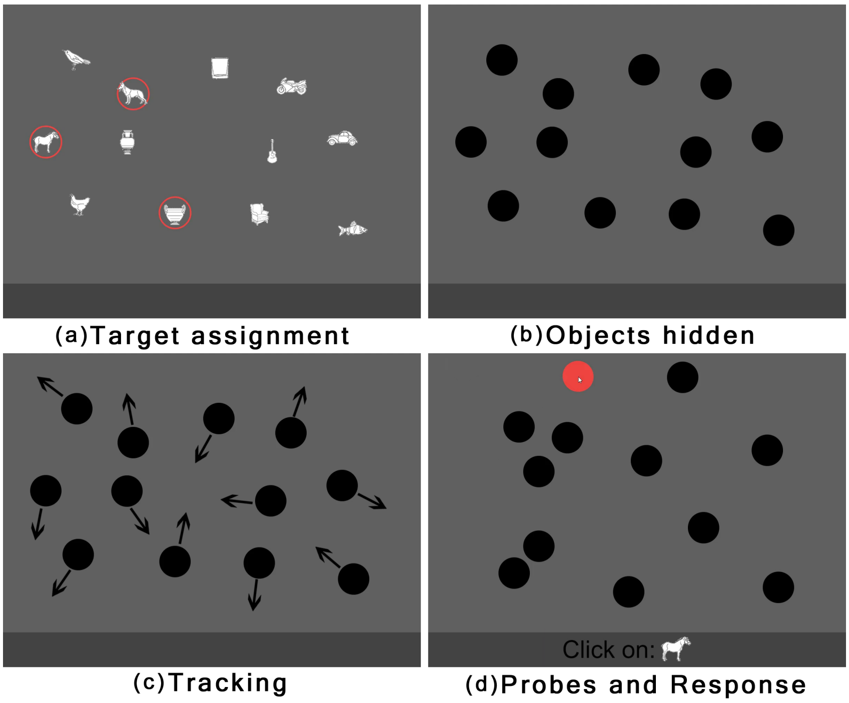

Figure 3. Illustration of a trial in Supplementary Experiment 2. First, targets were assigned by enclosing them in red circles (a), then all objects were hidden behind black disks (b) before they started moving around the display (c). Probes appeared at the bottom of the display during the response phase (d), where participants indicated the position of the probes.

Methods

Participants. We recruited 64 participants (15 females) with a mean age of 32.2 years (range: 18–59 years, *SD*: 9.84 years).

Apparatus. Identical to the other experiments in the main manuscript.

Stimuli. We used the same object images as in Experiment 1.

Procedure. This was similar to Experiment 3 in the main manuscript, except for the following: we used 3 target objects (balanced with 1 animal and 2 artifacts and 2 animals and 1 artifact) and objects were hidden behind black circles during the

tracking phase (see Figure 3). The circles turned red when clicked on during the response phase. The objects moved with a speed of 12 pixels per frame.

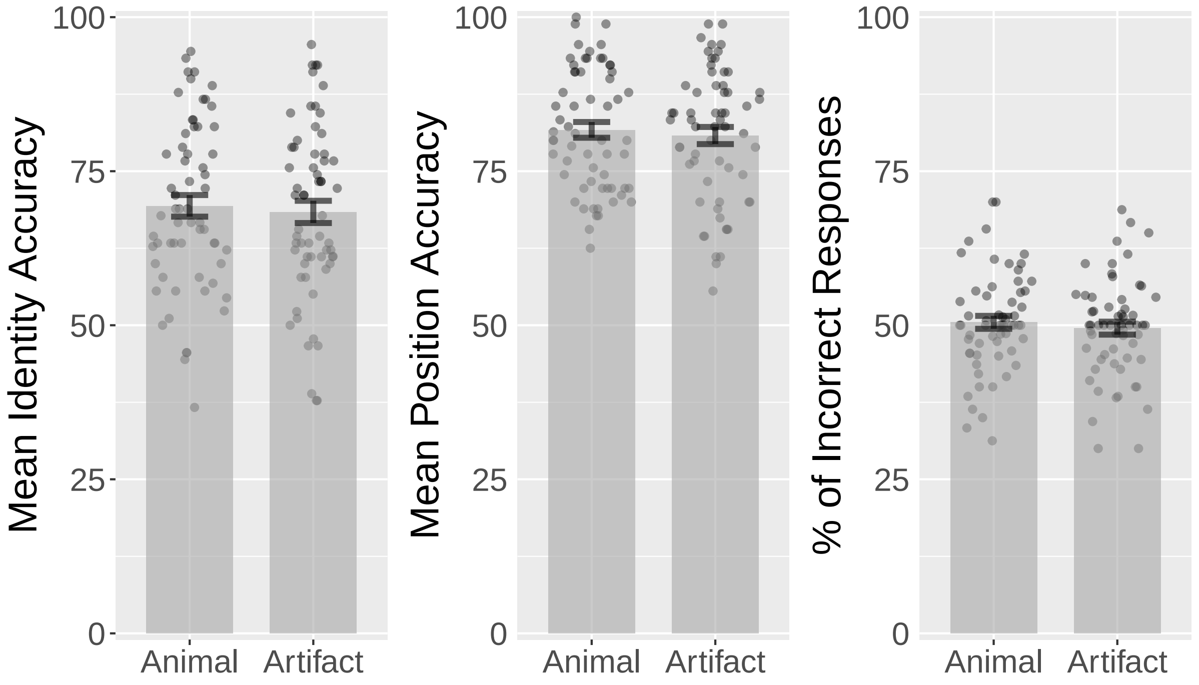

Figure 4. Combined bar and scatter plots for Supplementary Experiment 2 on mean identity accuracy, position accuracy and percentage of incorrect responses by category. Error bars show standard errors and the superimposed scatterplots show mean values from each participant. Identity accuracy shows how accurately participants could localize the individual targets after the tracking period. Position accuracy shows percentage correct localizations of targets, irrespective of their identities.

Results

Before conducting the statistical analysis we removed the data from 3 participants for having mean identity accuracy below 40% (1.5 *SD* below the median). A t-test on identity accuracy between animal (M = 69.4%, SD = 13.7%) and artifact targets (M = 68.4%, SD = 14.1%) showed no significant difference, $t(60) = 1.4$, $p = 0.17$, 95% CI [-0.44, 2.4], $d_z = 0.18$, $d_{rm} = 0.071$, $CL = 57\%$ (see Figure 4). A Bayesian t-test showed anecdotal evidence for the null hypothesis, $\mathrm{BF}_{01} = 2.908$.

Next, a t-test on position accuracy between animal (M = 81.7%, SD = 9.97%)

and artifact targets (M = 80.8%, SD = 10.9%) showed no significant difference, $t(60)$ = 1.5, p = 0.15, 95% CI [-0.35, 2.2], d_z = 0.19, d_{rm} = 0.087, CL = 57%. A Bayesian t-test showed anecdotal evidence for the null hypothesis, BF_{01} = 2.638.

Finally, before testing for a difference in percentage of incorrect responses, we removed 1 participant for only having one error. Animal distractors had a mean of 50.5% (SD = 8.1%), while artifact distractors had a mean of 49.5% (SD = 8.1%). The difference was however not significant, $t(59)$ = 0.45, p = 0.66, 95% CI [-3.3, 5.2], d_z = 0.058, d_{rm} = 0.12, CL = 52%. A Bayesian t-test showed moderate evidence for the null hypothesis, BF_{01} = 6.433.

Discussion

Requiring participants to explicitly encode target identities and to keep track of them during the tracking phase did not reveal significantly greater identity or position accuracies for animals compared to artifacts. Neither did it reveal a significant effect on the tendency to report animal distractors. The Bayes factors showed evidence in the same direction as the significance tests. However, they helped reveal that the evidence for the null hypothesis for the effect of category on identity and position accuracy was only anecdotal.

With respect to the objects not being visible during tracking, the reasoning for the present task was more in line with Experiment 1 in the main manuscript, with the exception of explicitly requiring the binding of identity to positions. In this manner, we attempted to measure if the association between an animal stimuli and a particular position would improve tracking performance without allowing participants to continuously update the binding visually.

Thus, requiring participants to bind object identities to positions during assignment only, did not bring about an attentional advantage for animals.

Supplementary Experiment 3

Previous research has shown that objects moving in a random, unpredictable manner can appear as animate and capture attention (Pratt, Radulescu, Guo, & Abrams, 2010). As the objects in our previous experiments changed directions at random, without encountering the edge of the display or other objects, it could be that all objects in the display were perceived as animate and thus subject to animate monitoring, irrespective of their appearance. This could then effectively have erased any effect of animal images as all objects were tracked with the same priority.

To investigate this possibility we modified Experiment 2 such that on any given trial, half of the animals and half of the artifacts would move in predictable, physical patterns, only changing directions with predictable angles when colliding with edges or other objects. The other half changed directions with random angles at randomly selected time points or when colliding.

From this setup we expected to find that objects moving with random motions would be tracked more successfully, be prioritized in responses and lead to more distractors being reported as targets, compared to objects with predictable motions. Importantly, if random directional changes can eliminate a bias for animal images, we would expect to observe interactions where animal images are biased when moving predictably, but not when moving randomly.

Methods

Participants. We recruited 49 participants (27 females) with a mean age of 26.4 years (range: 16–53 years, *SD*: 7.98 years).

Apparatus. Identical to the other experiments in the main manuscript.

Stimuli. We used the same object images as in Experiment 2.

Procedure. This was similar to Experiment 2 in the main manuscript except that half of the objects moved in a predictable manner by changing directions in a specular manner when colliding the outer edge of the scene or other objects (the angle of incidence equaled the angle of reflection). The other half could change directions at any moment or when colliding with other objects, in addition, the angle of change were selected at random, making the results of collisions unpredictable for observers (which is identical to how objects moved in all previous experiments).

Results

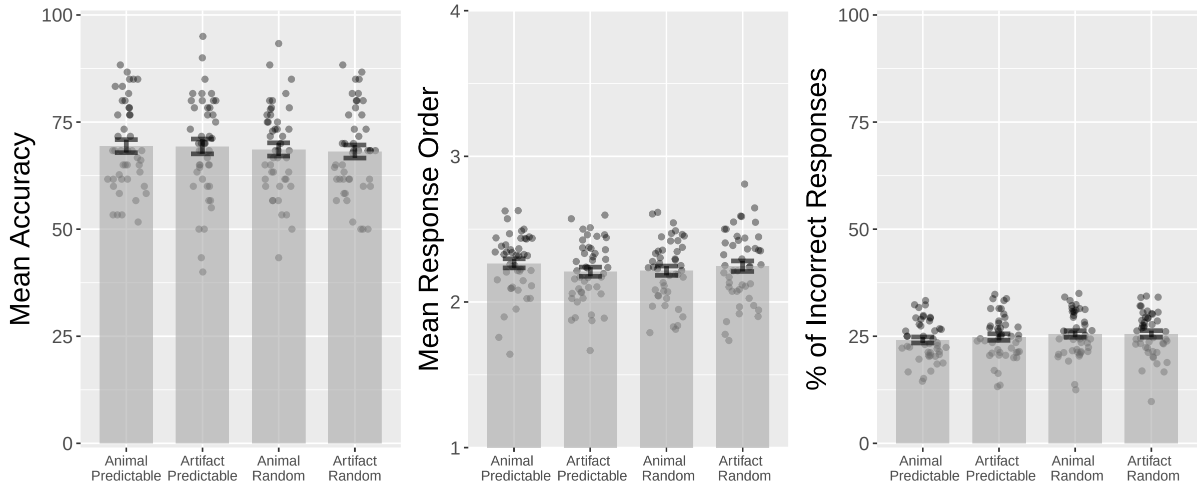

Figure 5. Combined bar and scatter plots for Supplementary Experiment 3 on mean accuracy, response orders and percentage of incorrect responses by category and motion type. Error bars show standard errors and the superimposed scatterplots show mean values from each participant.

Before performing the statistical analysis we removed 4 participants for having mean accuracy below 50% (1.5 *SD* below the median). An ANOVA on accuracy over target category (animal, artifact) and motion type (predictable, random), revealed a non-significant main effect of target category, $F(1, 44) = 0.05$, $p = .82$, $\eta_p^2 < .01$, $\eta_g^2 <$

.01. The effect of motion type was also non-significant, $F(1, 44) = 0.86$, $p = .36$, $\eta_p^2 = .02$, $\eta_g^2 < .01$, The interaction was also non-significant, $F(1, 44) = 0.06$, $p = .81$, $\eta_p^2 < .01$, $\eta_g^2 < .01$ (see Figure 5). Across conditions, animal targets had a mean accuracy of 68.9% (SD = 10.2%), while artifact targets had a mean accuracy of 68.7% (SD = 10.8%). Targets with predictable motions had a mean accuracy of 69.3% (SD = 10.8%), while targets with random motions had a mean accuracy of 68.3% (SD = 10.2%). A Bayesian repeated measures ANOVA on accuracy revealed moderate evidence for the null hypothesis of target category, $\text{BF}_{01} = 6.1$, motion type, $\text{BF}_{01} = 4.2$, and their interaction $\text{BF}_{01} = 4.5$.

An ANOVA on response orders over target category (animal, artifact) and motion type (predictable, random), revealed a non-significant main effect of target category, $F(1, 44) = 0.23$, $p = .63$, $\eta_p^2 < .01$, $\eta_g^2 < .01$. The effect of motion type was also non-significant, $F(1, 44) = 0.05$, $p = .82$, $\eta_p^2 < .01$, $\eta_g^2 < .01$. As was the interaction, $F(1, 44) = 2.53$, $p = .12$, $\eta_p^2 = .05$, $\eta_g^2 = .01$. Animal targets had a mean of 2.24 (SD = 0.21), while artifact targets had a mean of 2.22 (SD = 0.22). Targets with predictable motions had a mean of 2.23% (SD = 0.21%), while targets with random motions had a mean of 2.23% (SD = 0.22%). A Bayesian repeated measures ANOVA on response orders revealed moderate evidence for the null hypothesis of target category, $\text{BF}_{01} = 5.7$ and motion type, $\text{BF}_{01} = 5.6$. The evidence for the null hypothesis of the interaction was anecdotal and inconclusive, $\text{BF}_{01} = 1.5$.

An ANOVA on percentages of incorrect responses over target category (animal, artifact) and motion type (predictable, random), revealed a non-significant main effect of target category, $F(1, 44) = 0.16$, $p = .69$, $\eta_p^2 < .01$, $\eta_g^2 < .01$. The effect of motion type was also non-significant, $F(1, 44) = 1.39$, $p = .24$, $\eta_p^2 = .03$, $\eta_g^2 = .01$. As was the interaction, $F(1, 44) = 0.15$, $p = .69$, $\eta_p^2 < .01$, $\eta_g^2 < .01$. Animal distractors had a mean of 24.8% (SD = 4.9%), while artifact distractors had a mean of 25.1% (SD = 5.1%). Distractors with predictable motions had a mean of 24.4% (SD = 4.9%), while targets with random motions had a mean of 25.5% (SD = 5.1%). A Bayesian repeated measures ANOVA on percentages of incorrect responses revealed moderate evidence for

the null hypothesis of target category, $BF_{01} = 5.8$. The evidence for the null hypothesis of motion type was only anecdotal, $BF_{01} = 2.2$. Finally, the evidence for the null hypothesis of the interaction was moderate, $BF_{01} = 4.3$.

Discussion

We failed to find support for the prediction that objects moving with random motions should be tracked more successfully, be prioritized in responses and lead to more distractors being reported as targets, compared to objects with predictable motions. Importantly, we found moderate evidence for no interactive effects between image category and type of motion in tracking accuracy and percentages of incorrect responses. This suggests that the type of motion had little or no effect on revealing a bias for animal images. For response orders we only found anecdotal evidence for the null hypothesis, thus we cannot conclude on whether type of motion can influence an animal bias for this measure. However, response orders for predictable and random motions were highly similar, and thus do not fit the prediction of a general priority for objects with random motions, which should have been the causal mechanism responsible for abolishing a bias for animal images.

Combined analysis of experiment 5A and 5B

To increase power and get a better understanding of how the results looked across experiments, given that the experiments had identical procedures and varied only in the sets of stimuli, we combined the accuracy scores from both experiments in an ANOVA with Experiment (5A, 5B) and Category (animal, artifact) and Load (1,2). This analysis showed significant main effects of Experiment, $F(1, 110) = 7.75$, $p = .006$, $\eta_p^2 = .07$, $\eta_g^2 = .03$, Category, $F(1, 110) = 20.53$, $p < .001$, $\eta_p^2 = .16$, $\eta_g^2 = .03$, and Load, $F(1, 110) = 330.22$, $p < .001$, $\eta_p^2 = .75$, $\eta_g^2 = .48$. The interaction between Category and Load was significant, $F(1, 110) = 16.20$, $p < .001$, $\eta_p^2 = .13$, $\eta_g^2 = .02$. The interaction between Experiment and Category was not significant, $F(1, 110) = 0.02$, $p = .903$, $\eta_p^2 < .01$, $\eta_g^2 < .01$. The interaction between Experiment and Load was significant, $F(1, 110) = 6.79$,

p = .010, η_p^2 = .06, η_g^2 = .02. The interaction between Experiment, Category and Load was not significant, F(1, 110) = 0.84, p = .362, η_p^2 < .01, η_g^2 < .01. A Bayesian repeated measures ANOVA revealed anecdotal evidence for the alternate hypothesis of Category, BF_{01} = 0.42, and extreme evidence for Load, $\mathrm{BF}_{01} = 1.6 \times 10^{-66}$.

Next we conducted the same analysis on RTs and the results showed no significant effect of Experiment, F(1, 110) = 0.86, p = .355, η_p^2 < .01, η_g^2 < .01, but significant effects of Category, F(1, 110) = 11.64, p < .001, η_p^2 = .10, η_g^2 = .01, and Load, F(1, 110) = 371.47, p < .001, η_p^2 = .77, η_g^2 = .36. The interaction between Category and Load was not significant, F(1, 110) = 0.56, p = .456, η_p^2 < .01, η_g^2 < .01. Neither was any of the other interactions; between Experiment and Category, F(1, 110) = 5.39, p = .022, η_p^2 = .05, η_g^2 < .01, between Experiment and Load, F(1, 110) < 0.01, p = .970, η_p^2 < .01, η_g^2 < .01, between Experiment, Category and Load, F(1, 110) = 0.55, p = .460, η_p^2 < .01, η_g^2 < .01. A Bayesian repeated measures ANOVA revealed anecdotal evidence for the alternate hypothesis of Category, BF_{01} = 0.93, and extreme evidence for Load, $\mathrm{BF}_{01} = 2.4 \times 10^{-63}$.

Combined analysis of experiment 6A and 6B

To increase power and get a better understanding of how the results looked across experiments we analyzed the combined experiments. An ANOVA on accuracy showed a significant effect of Load, F(1, 124) = 999.69, p < .001, η_p^2 = .89, η_g^2 = .66. None of the other effects reached significance; Experiment, F(1, 124) = 1.79, p = .183, η_p^2 = .01, η_g^2 < .01, Category F(1, 124) = 1.08, p = .300, η_p^2 < .01, η_g^2 < .01, and interactions between Category and Load, F(1, 124) < 0.01, p = .983, η_p^2 < .01, η_g^2 < .01, between Experiment and Category, F(1, 124) = 0.32, p = .571, η_p^2 < .01, η_g^2 < .01, between Experiment and Load F(1, 124) = 1.82, p = .179, η_p^2 = .01, η_g^2 < .01, between Experiment, Category and Load, F(1, 124) = 0.71, p = .402, η_p^2 < .01, η_g^2 < .01. A Bayesian repeated measures ANOVA revealed moderate evidence for the null hypothesis of Category, BF_{01} = 9.2, and extreme evidence for Load, $\mathrm{BF}_{01} = 5.9 \times 10^{-124}$.

Next we conducted the same analysis on RTs, which showed a significant effect of

Load, $F(1, 124) = 168.10$, $p < .001$, $\eta_p^2 = .58$, $\eta_g^2 = .11$. None of the other effects reached significance; Experiment, $F(1, 124) = 0.75$, $p = .389$, $\eta_p^2 < .01$, $\eta_g^2 < .01$, Category, $F(1, 124) = 0.28$, $p = .596$, $\eta_p^2 < .01$, $\eta_g^2 < .01$, interaction between Category and Load, $F(1, 124) = 0.02$, $p = .875$, $\eta_p^2 < .01$, $\eta_g^2 < .01$, between Experiment and Category, $F(1, 124) = 0.93$, $p = .337$, $\eta_p^2 < .01$, $\eta_g^2 < .01$, between Experiment and Load, $F(1, 124) = 0.53$, $p = .470$, $\eta_p^2 < .01$, $\eta_g^2 < .01$, between Experiment, Category and Load, $F(1, 124) = 0.10$, $p = .751$, $\eta_p^2 < .01$, $\eta_g^2 < .01$. A Bayesian repeated measures ANOVA revealed moderate evidence for the null hypothesis of Category, $BF_{01} = 9.1$, and extreme evidence for Load, $BF_{01} = 5.3 \times 10^{-24}$.

References

Pratt, J., Radulescu, P. V., Guo, R. M., & Abrams, R. A. (2010). It's alive! animate motion captures visual attention. *Psychological Science*, *21*(11), 1724–1730.
